# Analysis of the role of GSK3 in the mitotic checkpoint

**DOI:** 10.1038/s41598-018-32435-w

**Published:** 2018-09-24

**Authors:** M. S. Rashid, T. Mazur, W. Ji, S. T. Liu, W. R. Taylor

**Affiliations:** Department of Biological Sciences, University of Toledo, 2801 W. Bancroft Street, MS601, Toledo, OH 43606 USA

## Abstract

The mitotic checkpoint ensures proper chromosome segregation; defects in this checkpoint can lead to aneuploidy, a hallmark of cancer. The mitotic checkpoint blocks progression through mitosis as long as chromosomes remain unattached to spindle microtubules. Unattached kinetochores induce the formation of a mitotic checkpoint complex (MCC) composed of Mad2, BubR1, Bub1 and Bub3 which inhibits anaphase onset. Spindle toxins induce prolonged mitotic arrest by creating persistently unattached kinetochores which trigger MCC formation. We find that the multifunctional ser/thr kinase, glycogen synthase kinase 3 (GSK3) is required for a strong mitotic checkpoint. Spindle toxin-induced mitotic arrest is relieved by GSK3 inhibitors SB 415286 (SB), RO 318220 (RO) and lithium chloride. Similarly, targeting GSK3β with knockout or RNAi reduced mitotic arrest in the presence of Taxol. GSK3 was required for optimal localization of Mad2, BubR1, and Bub1 at kinetochores and for optimal assembly of the MCC in spindle toxin-arrested cells. The WNT- and PI3K/Akt signaling pathways negatively regulate GSK3β activity. Inhibition of WNT and PI3K/Akt signaling, in the presence of Taxol, induced a longer mitotic arrest compared to Taxol alone. Our observations provide novel insight into the regulation of the mitotic checkpoint and its connection to growth-signaling pathways.

## Introduction

The mitotic checkpoint monitors attachment of chromosomes to spindle microtubules and blocks anaphase onset until all the chromosomes attain bi-orientation^1–3^. A defining feature of cancer cells is having fewer or more than 2 copies of each chromosome and/or chromosomal segment, typically called aneuploidy. Imbalances in chromosome number may contribute to overexpression of oncogenes or loss of tumor suppressor loci. Additionally, chromosome mis-segregation produces cytosolic DNA which triggers the GAS-STING inflammatory pathway which drives metastasis^4,5^. Increased rates of chromosome mis-segregation, called chromosome instability (CIN), has been proposed to occur in part from mutations in mitotic checkpoint genes during cancer progression^6–8^. The origins and consequences of aneuploidy and CIN in cancer are not completely understood^9^.

The mitotic checkpoint is composed of a group of evolutionarily conserved proteins including Mad1, Mad2, BubR1, Bub1, Bub3 and Mps1. These proteins localize to unattached kinetochores, where they generate the mitotic checkpoint complex (MCC) consisting of Mad2, BubR1-Bub3 and Cdc20^3,10^. Aberrant attachments such as syntelic, where microtubules from both poles bind to the same kinetochore, or monotelic attachments, where microtubules from only one pole bind to one kinetochore, result in unattached kinetochores and can cause chromosome mis-segregation if allowed to proceed^10^. In response, the mitotic checkpoint generates MCC which inhibits the multisubunit E3 ubiquitin ligase Anaphase Promoting Complex/Cyclosome (APC/C) to prevent mitotic progression^3,11^. The APC/C contains a “destruction box” via which it targets Securin and Cyclin B for degradation by the proteasome^12^. Degradation of Securin allows sister-chromatid cohesion to dissolve, and degradation of Cyclin B inactivates CDK1 to promote mitotic exit. Thus, an active APC/C promotes chromosome disjunction and mitotic exit. Cdc20 is an APC/C co-activator and contains WD-40 domains which are bound by APC/C substrates. When the MCC binds the APC/C^Cdc20^, it shifts the position of the APC/C-bound Cdc20 preventing substrate recognition. In this manner, the MCC inhibits APC/C in response to mis-aligned chromosomes and prevents mitotic progression^13–17^. The chromosomal passenger complex (CPC) composed of INCENP, Survivin, Borealin and Aurora B kinase provides an additional layer to monitor proper chromosome attachment to the spindle^18^. In this case, CPC destabilizes inappropriate attachments of chromosomes to the spindle (for example, both kinetochores attached to microtubules from the same pole). This destabilization creates unattached kinetochores that activate the mitotic checkpoint^18^. CPC malfunction can lead to cytokinesis defects, chromosome congression and segregation defects, spindle checkpoint malfunction and improper spindle pole formation^19^.

Checkpoint activation involves the hierarchal recruitment of the mitotic checkpoint proteins to kinetochores to generate a catalytic platform. First, Mad1 and Bub1 are recruited to the kinetochores by Mps1 phosphorylation of Knl1 MELT repeats^20–22^. At the kinetochore Mad1 binds to open-Mad2 (o-Mad2) and catalyzes its refolding to an alternative tertiary conformation: closed-Mad2 (c-Mad2)^23,24^. Simultaneously, Bub1 recruits and stabilizes BubR1 at unattached kinetochores, where BubR1 binds Cdc20 in complex with c-Mad2^25^. Importantly, only c-Mad2 is incorporated into MCC. c-Mad2 and BubR1-Bub3 co-operatively inhibit Cdc20 substrate recognition by the APC/C to inhibit anaphase onset^11,24,26^.

Mammalian cells may exit mitosis in the presence of spindle toxins by several mechanisms. In mitotic slippage, a basal level of APC/C activity degrades Cyclin B below a threshold level whereupon the cells exit mitosis without satisfying the mitotic checkpoint^27,28^. Alternatively, mitotic exit may be due to a gradual decrease in MCC abundance during prolonged arrest. A “weakened” mitotic checkpoint may be caused by mutations in or reduced expression of the mitotic checkpoint proteins and this can translate to an accelerated rate of mitotic exit^29^. Mad2 localization at kinetochores correlates with mitotic checkpoint strength; decreased Mad2 levels at the kinetochores resulted in shorter mitotic duration times in the presence of spindle toxins^13,30,31^. Additionally, levels of Mad2-Cdc20 complex determines the rate of Cyclin B degradation and mitotic exit, with decreasing levels of Mad2-Cdc20 interactions translating to increased Cyclin B degradation^32^. Understanding how mitotic checkpoint strength is modulated, and how key players such as Mad2 are regulated in this modulation, has implications for the clinical effectiveness of spindle toxins.

GSK3 contributes to a plethora of biological processes functioning downstream of the WNT pathway and responsive to Akt-dependent signaling pathways^33^. Both WNT-signaling and Akt signaling activity peak during mitosis, and WNT plays a clear role in spindle dynamics as well as modulating mitotic protein levels^34,35^. GSK3 regulates the G1/S transition by affecting Cyclin D expression via β-catenin^36^. When WNT ligand is present, GSK3β is inhibited from phosphorylating its target β-catenin in the cytoplasm. This allows β-catenin to accumulate and translocate to the nucleus where it induces transcription of TCF/LEF-target genes including Cyclin D and c-MYC, which promote cell proliferation^36^. When WNT ligand is absent, GSK3β phosphorylates β-catenin and targets it for ubiquitination and degradation^36^. GSK3 also plays a role in microtubule dynamics and spindle morphology^37,38^. In addition to the WNT pathway, GSK3 is phosphorylated and inhibited by Akt, placing GSK3 downstream of multiple tyrosine kinase receptor pathways. Interestingly, Akt localizes to spindle poles during mitosis^37^. Additional evidence shows that the PI3K/PTEN/Akt influences the spindle checkpoint. For example, PTEN deficient cells could not maintain spindle checkpoint activity when exposed to Taxol^37,39,40^. However, regulation of the mitotic checkpoint by WNT/GSK3 or PI3K/Akt/GSK3 signaling is poorly defined. A previous study showed that GSK3 inhibition increased chromosome mis-alignment, however, progression through mitosis was not halted^38^. These observations would be consistent with a requirement for GSK3 to establish the mitotic checkpoint, however this idea was not directly tested.

Our experiments show that multiple GSK3 kinase inhibitors induce mitotic exit in HeLa cells exposed to spindle toxins. The efficiency of exit was enhanced for cells that had already been blocked in mitosis for several hours suggesting that GSK3 contributes to the strength of the mitotic checkpoint but does not act as an on/off switch in this response. Since GSK3 acts a signaling hub, our results suggest that GSK3 may link the strength of the mitotic checkpoint with the external environment via multiple signal transduction pathways. Consistent with this idea, inhibiting either WNT or PI3K signaling increased mitotic arrest in spindle toxins. Overall, our study indicates that GSK3 regulates the strength of the mitotic checkpoint and potentially connects the PI3K and WNT-signaling pathways to mitosis.

## Results

### GSK3 inhibitors abrogate the mitotic checkpoint

**Figure 1:**
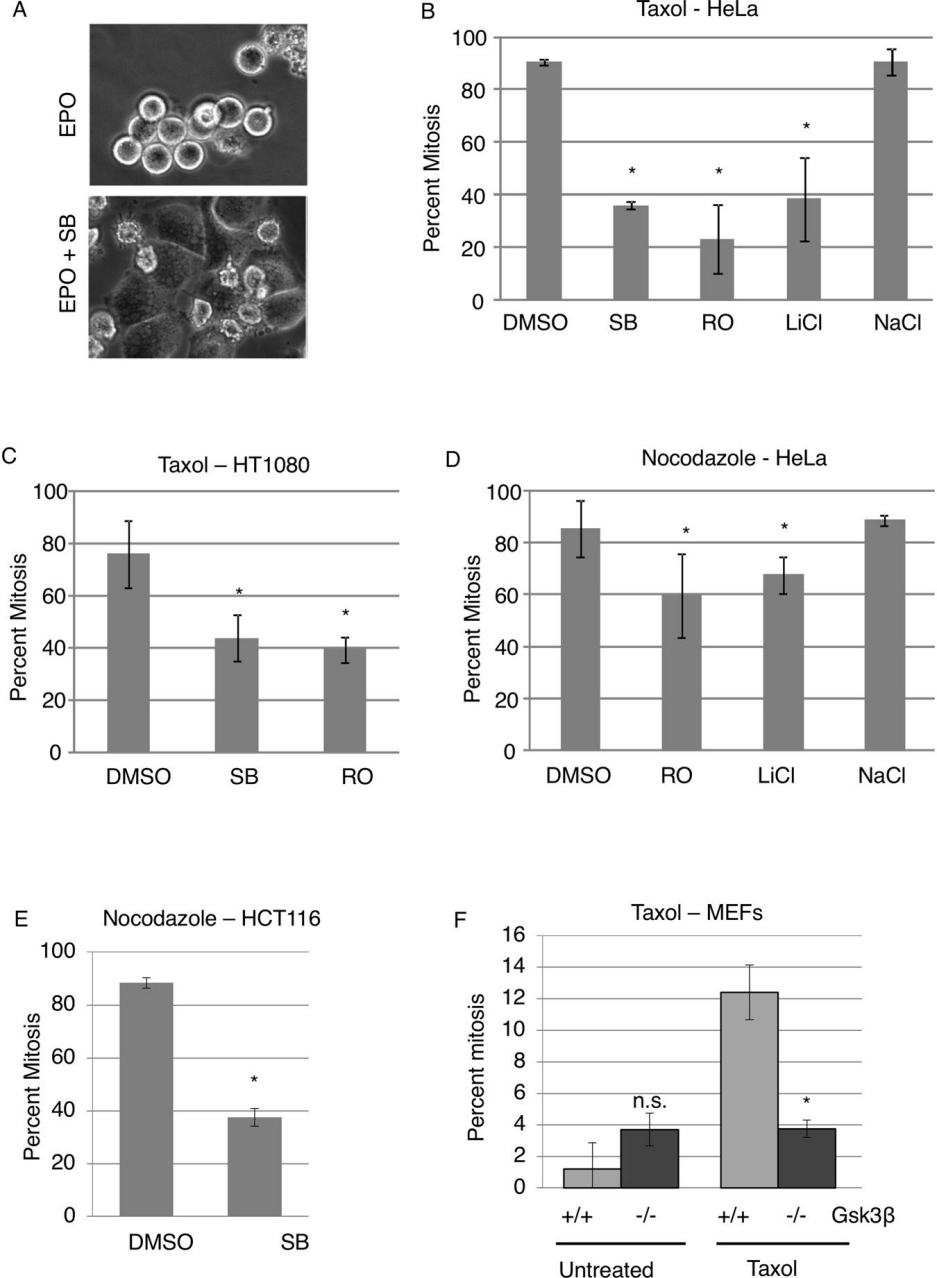
GSK3β inhibitors induce mitotic exit in the presence of spindle toxins. (**A**) HeLa cells were exposed to 20 nM epothilone B (EPO) for 24 hours. The GSK3 inhibitor, SB415286 (SB), was then added and the cells imaged 16 hours later. (**B**–**E**) Taxol (1 μM) or Nocodazole (3.3 μM) was added to Hela, HT1080, or HCT116 cells for 12–14 hours. GSK3 inhibitors and DMSO or NaCl controls were added for 2.5 hours and then mitotic index determined in a blinded manner using chromosome spreads. GSK3 inhibitors: SB, 25 μM SB415286; RO, 1 μM RO318220; 20 mM LiCl. Bars represent standard deviation. ^*^p < 0.05 using a student’s t-test. (**F**) HeLa M cells were exposed to Taxol(1 μM) for 12–14 hours, followed by 2 hours of MG132 and SB or RO or DMSO control. (**G**) MEFs from Gsk3β-null embryos or wild-type littermates were exposed to Taxol for 14 hours and mitotic index determined using chromosome spreads. (**B**–**F**) Experiments were repeated in triplicate with at least 100 cells measured per repeat. Error bars represent standard deviation. Standard deviation was calculated using triplicates from a single repeat. TTest analysis was performed to confirm if the difference was significant. * indicates p < 0.05.

A small library of kinase inhibitors was added to epothilone B (EPO)-arrested cells to observe their effects on mitosis. EPO triggers the mitotic checkpoint by stabilizing microtubules similarly to Taxol. Our initial visual assessment indicated that GSK3 inhibitors, SB 415286 (SB), RO 318220 (RO) and LiCl, released HeLa M cells from EPO-induced mitotic arrest (our unpublished data) (examples shown in Fig. 1A). Next mitotic index was measured in a blinded manner in multiple cell lines, in the presence of two different spindle toxins and the three GSK3 inhibitors. The cells were exposed to Taxol or Nocodazole for 12 hours, followed by 2.5 hours of SB, RO or LiCl. Chromosome spreads were performed to obtain the mitotic index. Chromosomes condense and nuclear envelope breaks down when cells enter mitosis and sister chromatids can be seen distinctly using Giemsa in chromosome spread analysis. When cells are in interphase, chromosomes are decondensed and nuclear envelope intact, the cell is seen with its intact nucleus and no distinct condensed chromosomes using Giemsa. (Supplemental Fig. 10B). GSK3 inhibitor treated cells showed a decreased mitotic index in the presence of either Taxol or Nocodazole compared to DMSO and NaCl control. (Fig. 1B–E) Western blot showed GSK3β substrate β-catenin levels increased in SB or LiCl cells in Taxol-arrested cells, showing the inhibitors decrease GSK3β activity (Supplemental Fig. 1). To determine if the effect on mitotic arrest was due to GSK3 specifically we used several additional systems. First, mouse embryo fibroblasts (MEFs) from GSK3β-knockout mice were analyzed. Secondly, we used CRISPR to target GSK3β, in human cells, however, as described below we were unable to obtain stable GSK3β knockout human cells. Compared to wild-type MEFs, GSK3β-knockout MEFs show decreased mitotic arrest when exposed to Taxol. The knockout cells show a similar mitotic index as wild-type MEFs in the absence of Taxol (Fig. 1F). These observations indicate that GSK3β is required for operation of the mitotic checkpoint.

In our attempt to target GSK3β in HeLa cells we used two gRNAs designed against different exons of GSK3β. One gRNA was expressed from a plasmid that also expresses GFP-Cas9. The second gRNA was expressed from a plasmid also expressing a puromycin resistance and Cas9^41^. Both plasmids were co-transfected into HeLa M cells. GFP fluorescence was used to monitor transfection efficiency and puromycin selection was used to obtain single colonies. We observed some large multinucleated colonies mixed with the normal HeLa cell morphologies (data not shown). Clones with multinucleated cells grew slowly and in only in a few cases were we able to obtain enough cells for western analysis. Western blotting showed that cells with normal morphologies still expressed GSK3β. In other clones, GSK3β was gone suggesting that our CRISPR approach worked, but that unfortunately HeLa cells could not survive long-term without this protein (Supplemental Fig. 2A). As an alternative, we carried out transient transfection with our CRISPR plasmids. Western blotting showed decreased GSK3β in cells with GSK3β CRISPR, compared with IL-17 CRISPR transfected cells. The partial response is likely due to the heterogeneity of knockout in the transient setting. In this case, we observed fewer cells arrested in mitosis with Taxol after transient transfection with gRNAs targeting GSK3β (Supplemental Fig. 2B).

### GSK3 regulates the strength of the mitotic checkpoint

**Figure 2:**
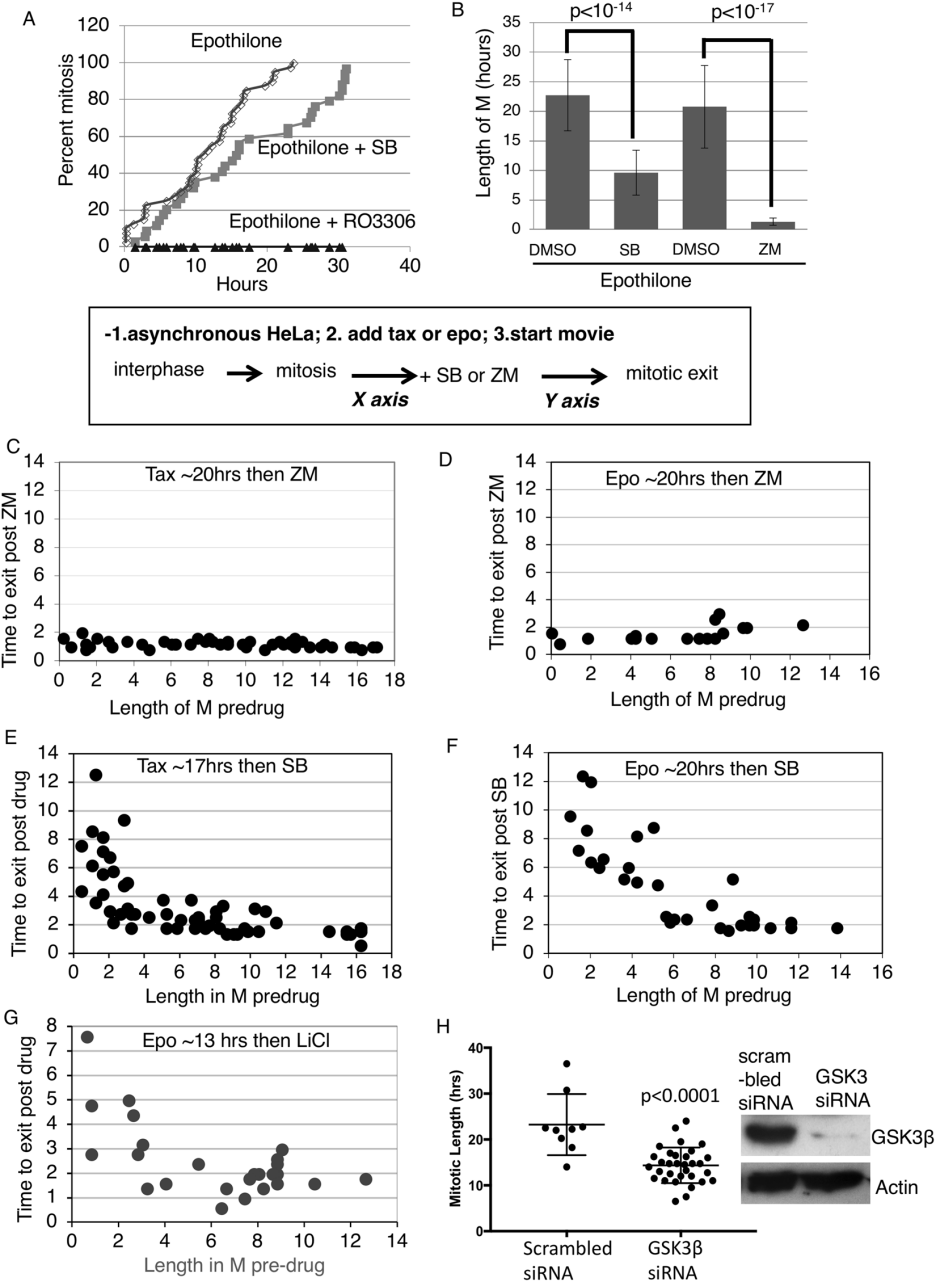
GSK3 regulates the strength of the mitotic checkpoint. (**A**,**B**) HeLa cells exposed to 20 nM EPO with or without SB, ZM447439 (ZM), or CDK1 inhibitor RO3306 were tracked by phase contrast microscopy for mitotic entry and exit. (**C**–**G**) Asynchronous HeLa cells were tracked upon addition of Taxol or EPO by live cell imaging using phase contrast microscopy. GSK3 inhibitors, SB415286 or LiCl, or Aurora B inhibitor, ZM, was added and the live cell imaging continued to follow the same cells. The length of time before and after mitosis entry and mitotic exit was correlated. (as indicated in the box). (**H**) Hela cells were transfected with GSK3β RNAi or with scrambled siRNA. Cells were exposed to 1 μM Taxol 3-days post transfection and time lapse imaging used to analyze mitotic length. ~30 cells were analyzed per experiment. Western blot (WB) was done for GSK3β and Actin loading control, to show knockdown of GSK3β. siRNA against human GSK3β or scrambled siRNA was transfected into HeLa cells and 3 days post-transfection cells were subjected to WB analysis.

Cells in a prolonged mitotic arrest induced by antimitotic drugs must ultimately accept one of two fates: either they will die by the apoptotic pathway or undergo mitotic exit. Mitotic exit can occur due to a gradually weakened checkpoint or lead to mitotic slippage in which case cells exit mitosis in the presence of an intact mitotic checkpoint^42^. To further characterize the effects of GSK3 inhibition, we followed cells by time-lapse microscopy. Representative images showing mitotic entry and exit in time-lapse imaging are shown (Supplemental Fig. 10A). As seen via phase-contrast, when the cells enter mitosis they round up, and keep this rounded form until the end of mitosis. When they enter interphase the cells flatten out and are not rounded (Supplemental Fig. 10A). Measurement of mitotic exit and entry via time-lapse imaging has been described in other studies^42^. HeLa cells were co-treated with EPO and SB and followed by time-lapse. We plotted the cumulative mitotic index in cells exposed to EPO in the presence or absence of SB. Addition of SB shifted the curve to the right indicating a ~50% delay in entering mitosis (Fig. 2A). The fact that ~100% of SB-treated cells eventually enter mitosis indicates that SB does not block cells in interphase under these conditions.

SB has been reported to inhibit Cdk2/Cyclin A suggesting that it may also inhibit Cdk1^43^. However, HeLa cells continuing to enter mitosis when exposed to SB suggests no direct effect on Cdk1/Cyclin B under the conditions of our assay (Fig. 2A). For comparison, adding the CDK1 inhibitor RO3306 completely blocked mitotic entry in cells also exposed to EPO (Fig. 2A). We also measured the mitotic phosphorylation of histone H1 (pH1) using immunofluorescence in cells exposed to SB. Mitotic phosphorylation of pH1 is catalyzed by Cdk1^44^. We observed that in the presence of Taxol, SB415285 did not affect pH1 (Supplemental Fig. 9). Some of our experiments used the GSK3 inhibitor RO 318220 which has also been reported to inhibit PKC. However, adding the PKC inhibitor Chelerythrine Chloride had no effect on the mitotic block induced by Taxol suggesting that potential off-target effects on PKC have little impact on the mitotic phenotype we observe (Supplemental Fig. 11).

Next, we used time-lapse microscopy to measure the length of mitosis in cells exposed to EPO with or without either SB or ZM447439 (ZM), an Aurora B inhibitor. As previously shown, inhibition of Aurora B significantly abrogated the checkpoint and cells exited mitotic block^42^ (Fig. 2B). SB significantly shortened the duration in mitosis in the presence of EPO but not as efficiently as ZM (Fig. 2B).

Aurora B is essential for the mitotic arrest induced by EPO and inspection of time-lapse movies shows nearly all cells exiting mitosis shortly after adding ZM (our unpublished data). In contrast, it appeared that after adding SB or LiCl to EPO-blocked cells, mitotic exit was heterogeneous with some cells exiting faster than others. To investigate this phenomenon, we followed both mitotic entry and exit by time lapse microscopy. Asynchronous cells were tracked by time lapse starting at the moment of adding EPO. The next day when most cells had entered mitosis, we added SB or LiCl and tracked the same cells for one more day. In this way, we could correlate how long the cell had been previously arrested in EPO with how long it took for SB or LiCl to induce mitotic exit. As a comparison, we carried out the same analysis with ZM.

We observed that ZM initiates a near synchronous mitotic exit irrespective of the length of the previous mitotic block (Fig. 2C,D). In contrast, SB –induced or LiCl induced mitotic exit depended on how long the cells had been blocked in mitosis, with increased time in mitosis resulting in a shorter time to exit (Fig. 2E–G). We next used time-lapse microscopy to test the effect of GSK3β RNAi on the mitotic block induced by taxol. We were not able to repeat the exact kinetic experiments shown in Fig. 2E–G which require acute addition of the GSK3 inhibitor. Nonetheless, GSK3 RNAi reduced the mitotic duration in cells exposed to taxol when compared to scrambled siRNA (Fig. 2H). In this experiment, Hela cells were transfected with GSK3β RNAi, and three days post-transfection the cells were exposed to Taxol and followed by time-lapse imaging. Western blot analysis showed the decrease in GSK3β levels 3 days post RNAi knockdown (Fig. 2H). These results suggest that GSK3 may play a role in regulating the strength of the mitotic checkpoint.

**Figure 3:**
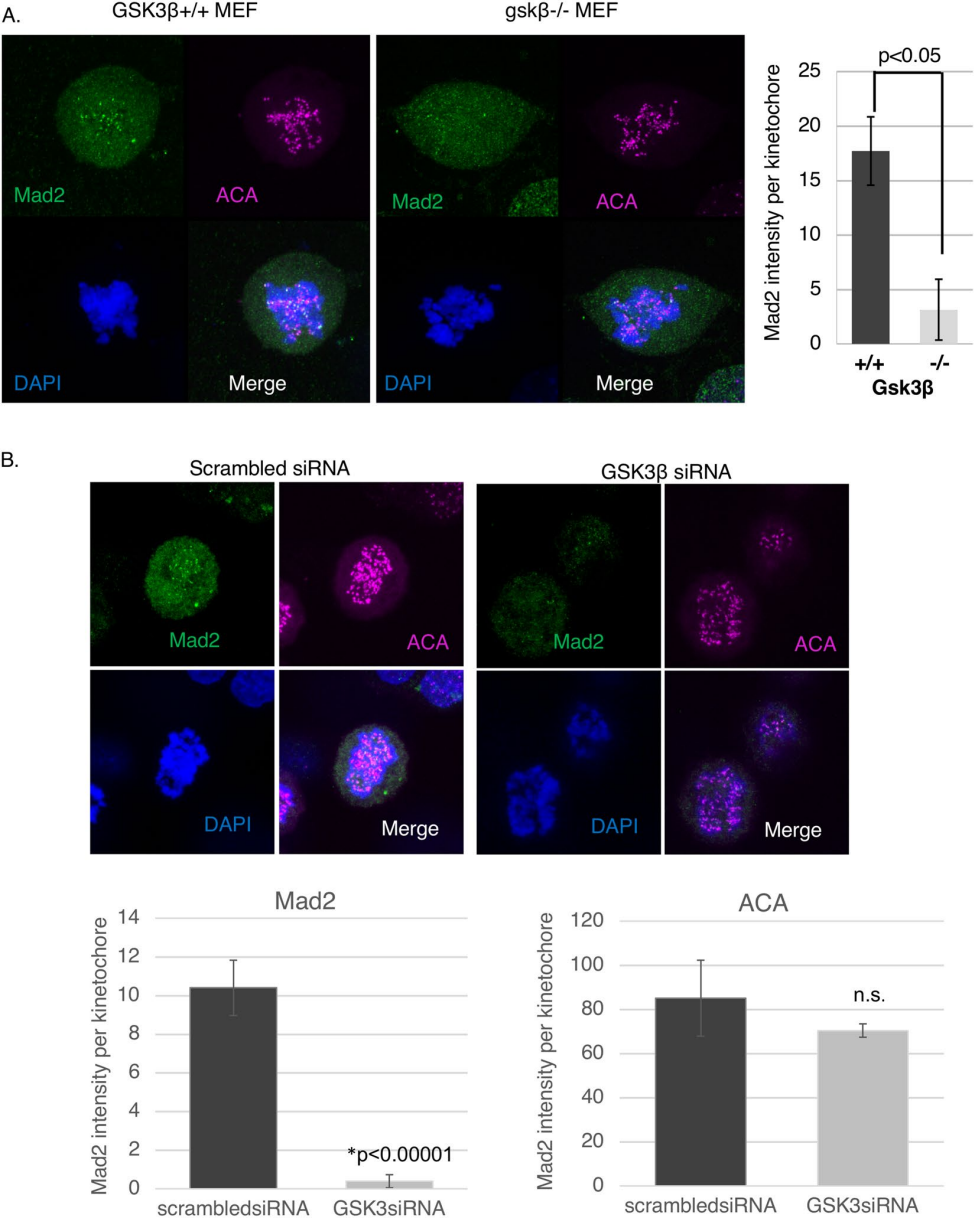
GSK3β knockout MEFs or GSK3β RNAi show decreased Mad2 levels at the kinetochores in presence of spindle toxins. (**A**) MEFs were exposed to Taxol for 12–14 hours, then immunofluorescence analysis performed to obtain Mad2 kinetochore intensity. ACA was used as a kinetochore marker. (**B**) siRNA against human GSK3β was transfected into HeLa cells. MG132 was added to inhibit the proteasome for 3 hours and subjected to immunofluorescence (IF) analysis. IF was performed to analyze Mad2 levels, ACA, a kinetochore marker, and DAPI to stain chromosomes. ~10 Cells per condition were imaged using confocal, using maximum projection images of z-stacks to analyze antigen levels at kinetochores. ~300 Kinetochores were analyzed for each condition, using ImageJ software to obtain intensity of the indicated antigens at kinetochores.

### GSK3 regulates Mad2 levels at kinetochores in mitosis

Next, we investigated the mechanism by which GSK3 regulates the mitotic arrest that occurs in spindle toxins. As indicated above, effects of SB on mitosis do not appear to be due to off-target effects on Cdk1/Cyclin B (Fig. 2A). Interestingly, cells exiting mitotic arrest in SB still attempted to generate cleavage furrows unlike ZM-treated cells (Supplemental Fig. 4A). Given that Aurora B helps to co-ordinate cleavage furrow ingression, these observations suggest that Aurora B may not be downstream of GSK3 in the mitotic checkpoint^45^. Consistent with this interpretation, levels of INCENP and Aurora B at kinetochores were unchanged by GSK3 inhibition (Supplemental Fig. 5; also see below). Aurora B phosphorylates histone 3 at serine 10 (pH3S10), which occurs during mitosis and is required for proper chromosome condensation and segregation. Mitotic histone H3S10 phosphorylation begins at prophase, and initiates at centromeres and progresses along the whole chromosome arm^18,46,47^. We investigated if SB has any effect on pH3S10 levels. Our data show that, in the presence of Taxol, pH3S10 levels remain unchanged with and without SB (Supplemental Fig. 9). These studies suggest that GSK3 may not target the CPC to maintain mitotic arrest.

**Figure 4:**
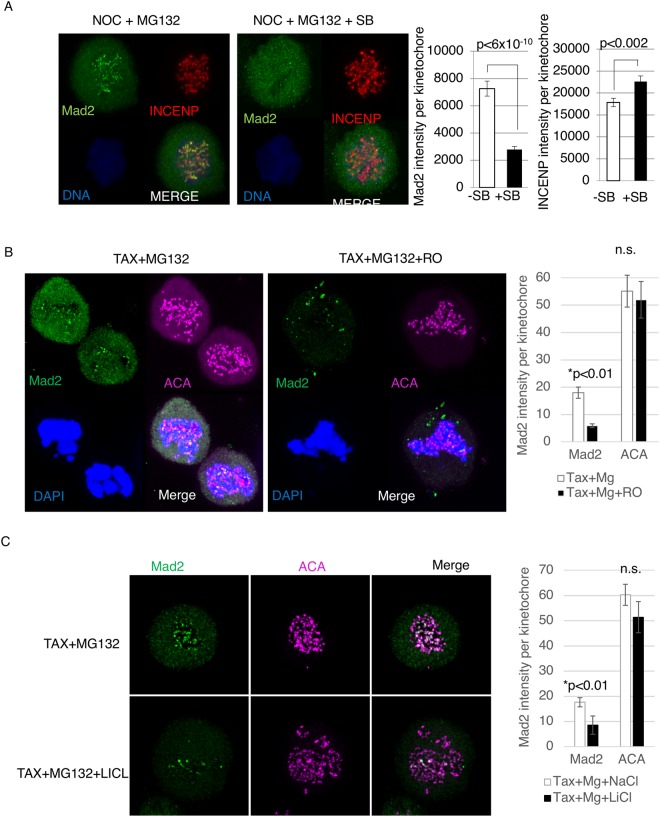
GSK3 inhibitors decrease Mad2 levels at the kinetochores in the presence of spindle toxins. (**A**) HeLa cells exposed to 2 μM Nocodazole with or without SB, were subjected to immunofluorescence analysis for Mad2, INCENP, and DAPI to stain chromosomes. MG132 was added to inhibit the proteasome. (**B**,**C**) HeLa cells exposed to 1 μM Taxol with or without RO (5 μM) or DMSO control, or LiCl (50 mM) or NaCl (50 mM) control. The cells were then subjected to immunofluorescence analysis for Mad2, ACA, and DAPI to stain chromosomes. MG132 was added to inhibit the proteasome. ~10 Cells per condition were imaged using confocal, using maximum projection images of z-stacks to analyze antigen levels at kinetochores. ~300 Kinetochores were analyzed for each condition, using ImageJ software to obtain intensity of the indicated antigens at kinetochores.

The level of Mad2 at the kinetochores is a well-defined marker of the operation of the mitotic checkpoint^13,30,31,48^. Interestingly, using various spindle poisons that induce varying levels of Mad2 association with the kinetochores, it was shown that the intensity of Mad2 kinetochores correlates with the time to mitotic exit^13^. As GSK3 appears to modulate the strength of the mitotic checkpoint, we investigated the effect of GSK3 inhibitors as well as GSK3*β* knockout on Mad2 kinetochore levels. Taxol was added to WT and *gsk3β−/−* MEFs for 12–14 hours. In these experiments, cells were also exposed to the proteasome inhibitor MG132 to block mitotic exit under conditions where the mitotic checkpoint was inactivated. *gsk3β−/−* MEFs showed decreased levels of Mad2 at kinetochores compared to wild type MEFs (Fig. 3A). Also, knocking down GSK3β using RNAi resulted in lower Mad2 accumulation to kinetochores in Taxol-treated cells (Fig. 3B). Next, Mad2 levels were analyzed by immunofluorescence in Taxol-arrested cells after adding SB, RO 318220 and LiCl and DMSO or NaCl as control. Under these conditions, SB, RO 318220 and LiCl decreased Mad2 levels at kinetochores compared to control (Fig. 4A–C). In contrast, SB had no effect on localization of INCENP to centromeres in taxol-treated cells (Fig. 4A).

### GSK3 regulates BubR1 and Bub1 levels at kinetochores in mitosis

**Figure 5:**
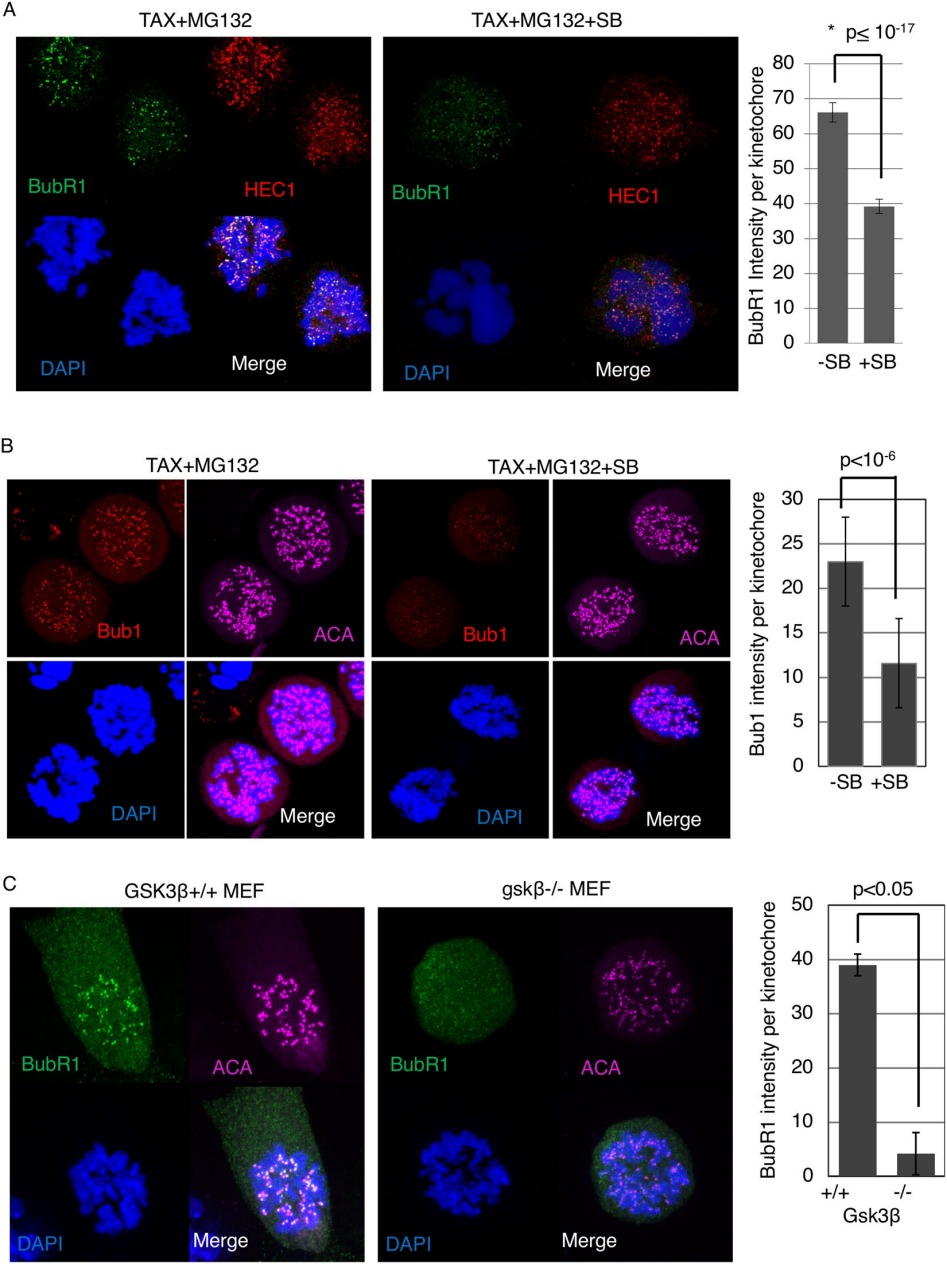
GSK3 inhibition decreases BubR1 and Bub1 levels at the kinetochores in the presence of spindle toxins. (**A**,**B**) HeLa cells exposed to 2 μM Nocodazole with or without SB, were subjected immunofluorescence analysis for BubR1 or Bub1 levels, ACA, a kinetochore marker, and DAPI to stain chromosomes. MG132 was added to inhibit the proteasome. (**C**) MEFs were exposed to Taxol for 12–14 hours, then immunofluorescence analysis performed to obtain BubR1 kinetochore intensity. ACA was used as a kinetochore marker. ~10 cells per condition were imaged using confocal, using maximum projection images of z-stacks to analyze kinetochores. ~300 kinetochores were analyzed for each condition.

Cells with an active mitotic checkpoint show high levels of BubR1 and Bub1 localized to kinetochores. BubR1 is assembled into the MCC along with Cdc20 and c-Mad2 generated at the kinetochores and a potent inhibition of APC/C activity depends on recruitment of BubR1-Bub3 to kinetochores^49^. Bub1 is required to recruit BubR1 and Mad1 at the kinetochores and is required for a functional mitotic checkpoint^50,51^. We investigated whether GSK3 could affect BubR1 and Bub1 levels at the kinetochores. Cells were exposed to Taxol for 12 hours following which SB and MG132 were added for 2.5 hours and immunofluorescence was carried out using antibodies to either BubR1 or Bub1. We observed reduced levels of BubR1 and Bub1 at kinetochores, while ACA, marking the kinetochores, remained constant (Fig. 5A,B). Similarly, when exposed to Taxol, *gsk3β−/−* MEFS showed reduced levels of BubR1 at kinetochores compared to wild-type MEFs (Fig. 5C).

The KMN network of proteins play essential roles in kinetochore structure and function. KMN is composed of the Knl1-complex, Ndc80 complex and Mis12-complex. Phosphorylation of Knl1 MELT-repeats creates docking sites for Bub3 and Bub1^52^. We investigated if GSK3 inhibition modulates two components of the KMN network. We analyzed the level of Knl1 and Hec1, an Ndc80 complex protein, in response to SB in the presence of Taxol. Knl1 and Hec1 levels remained unchanged in Taxol and SB co-treatment, compared to Taxol only, suggesting that GSK3 does not target these kinetochore proteins to regulate the mitotic checkpoint. (Supplemental Fig. 6, Fig. 5A).

### GSK3 increases MCC assembly in cells exposed to Taxol

**Figure 6:**
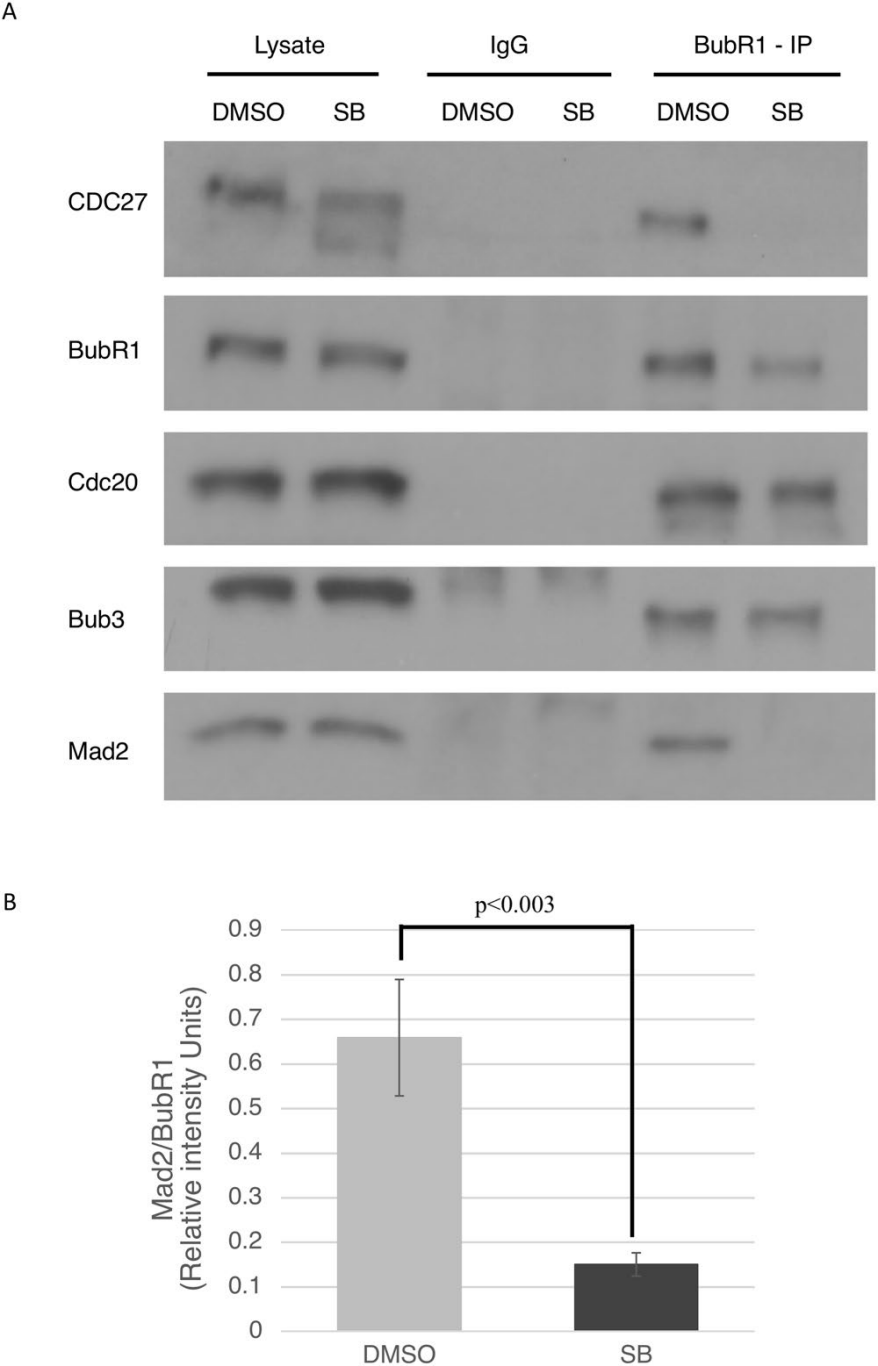
GSK3 inhibition decreases MCC assembly in the presence of spindle toxins. (**A**) HeLa cells were exposed to 1 μM Taxol for 12 hours, following which SB and MG132 was added. Cells were harvested after 2.5 hours and lysed. Co-immunoprecipitation was performed with extracted proteins pulled down using BubR1 antibody and IgG, as control, and 10% whole cell extract (“lysate”) was as used as input. (**B**) ImageJ was used to obtain pixel intensities of the bands. Mad2/BubR1 intensities were measured from three separate experiments.

Checkpoint proteins recruited to unattached kinetochores ultimately catalyze the formation of the MCC, composed of c-Mad2, Cdc20 and BubR1-Bub3. To test the effect of GSK3 on MCC formation, Taxol treated cells were exposed to SB and MG132 for 3 hours and cells were harvested and lysed. Cell lysates were immunoprecipitated with BubR1 antibodies. Immune complexes were analyzed by western blotting with antibodies against Mad2 to measure MCC levels. We observed that compared to DMSO control, no Mad2 was being pulled down with BubR1 in the SB-treated samples. BubR1 interaction with Cdc20 and Bub3 remained intact, indicating the GSK3 was not involved in these interactions (Fig. 6). Quantification from multiple experiments show that there was a ~75% decrease in the Mad2/BubR1 ratio in SB415286-treated cells (Fig. 6B). Quantification of Mad2/BubR1 binding in DMSO and SB treated samples in 3 separate experiments, show a decrease in Mad2 being pulled down with BubR1 in the presence of GSK3 inhibitors (Fig. 6, Supplemental Fig. 7).

### GSK3 overexpression enhances mitotic arrest

**Figure 7:**
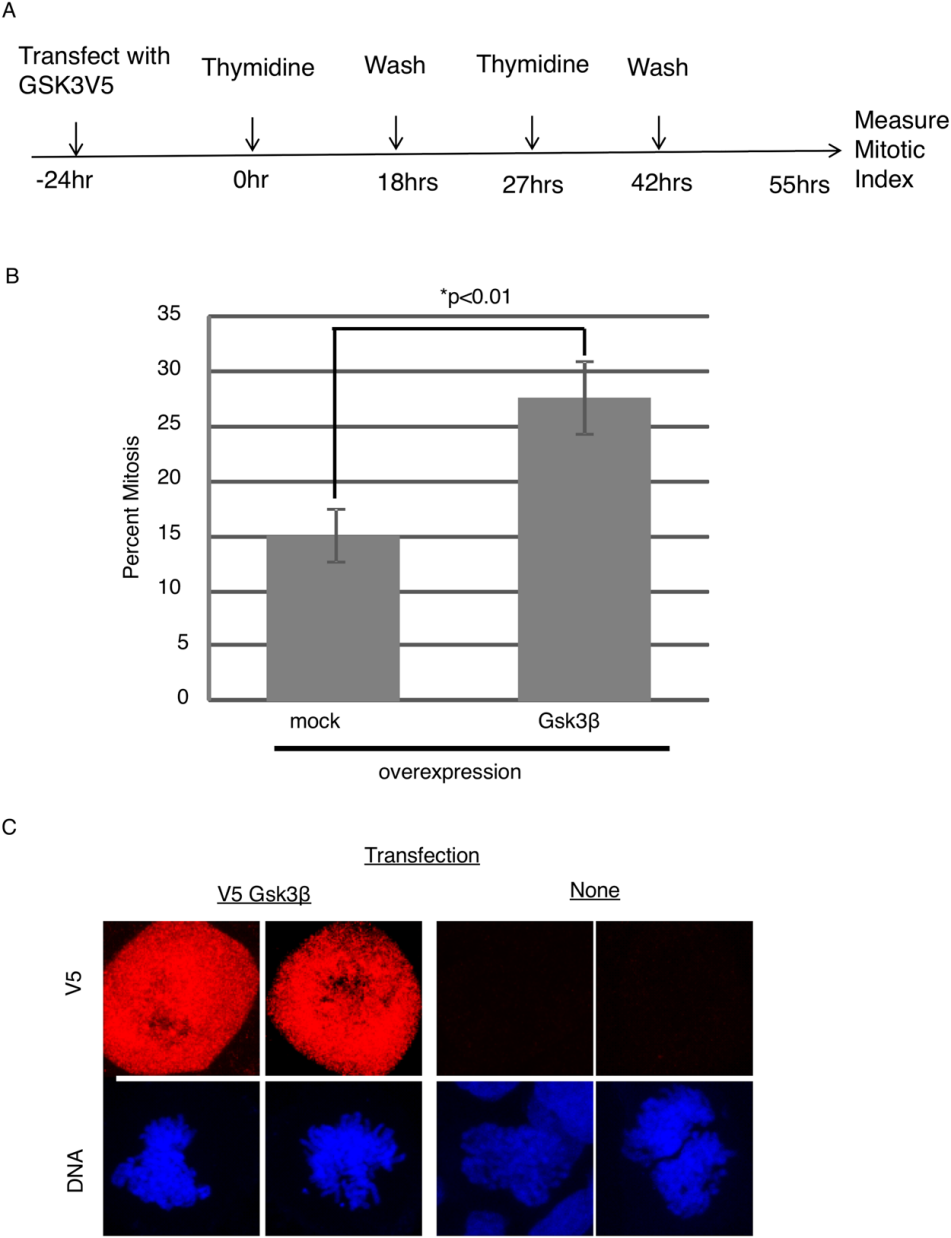
GSK3 overexpression induces a delay in mitotic exit. (**A**) HeLa cells were transfected with plasmid containing GSK3V5. Cells were subjected to double thymidine block and released for 13 hours, after which chromosome spreads were used to analyze mitotic index. ~300 cells were analyzed in a blinded study from each condition, which were in triplicate. (**B**) HeLa cells were transiently transfected with V5-Gsk3β and analyzed by immunofluorescence using V5 antibodies to confirm expression.

Our experiments indicate that MCC assembly and localization are regulated by GSK3. Tighe *et al*., have shown that GSK3 inhibition causes misalignment of chromosomes, and have attributed these affects to the role of GSK3 in microtubule dynamics. In contrast, these authors did not report on the effects of GSK3 inhibitors in cells blocked in mitosis with spindle toxins^38^. To better understand the role of GSK3 in mitotic regulation we tested the effect of overexpressing GSK3 protein in the absence of spindle toxins. Cells were transfected with a plasmid expressing V5-tagged GSK3β. Transfected cells were released from a double thymidine block and mitotic index determined 13 h later (Fig. 7A). Cells transfected with V5-tagged GSK3β showed a significantly higher mitotic index than untransfected cells consistent with a role for GSK3 in regulating either entry into or progress through mitosis (Fig. 7B). Expression of the transgene was confirmed by immunofluorescence (Fig. 7C).

### Inhibition of the WNT and PI3K/Akt pathways may strengthen the mitotic checkpoint

**Figure 8:**
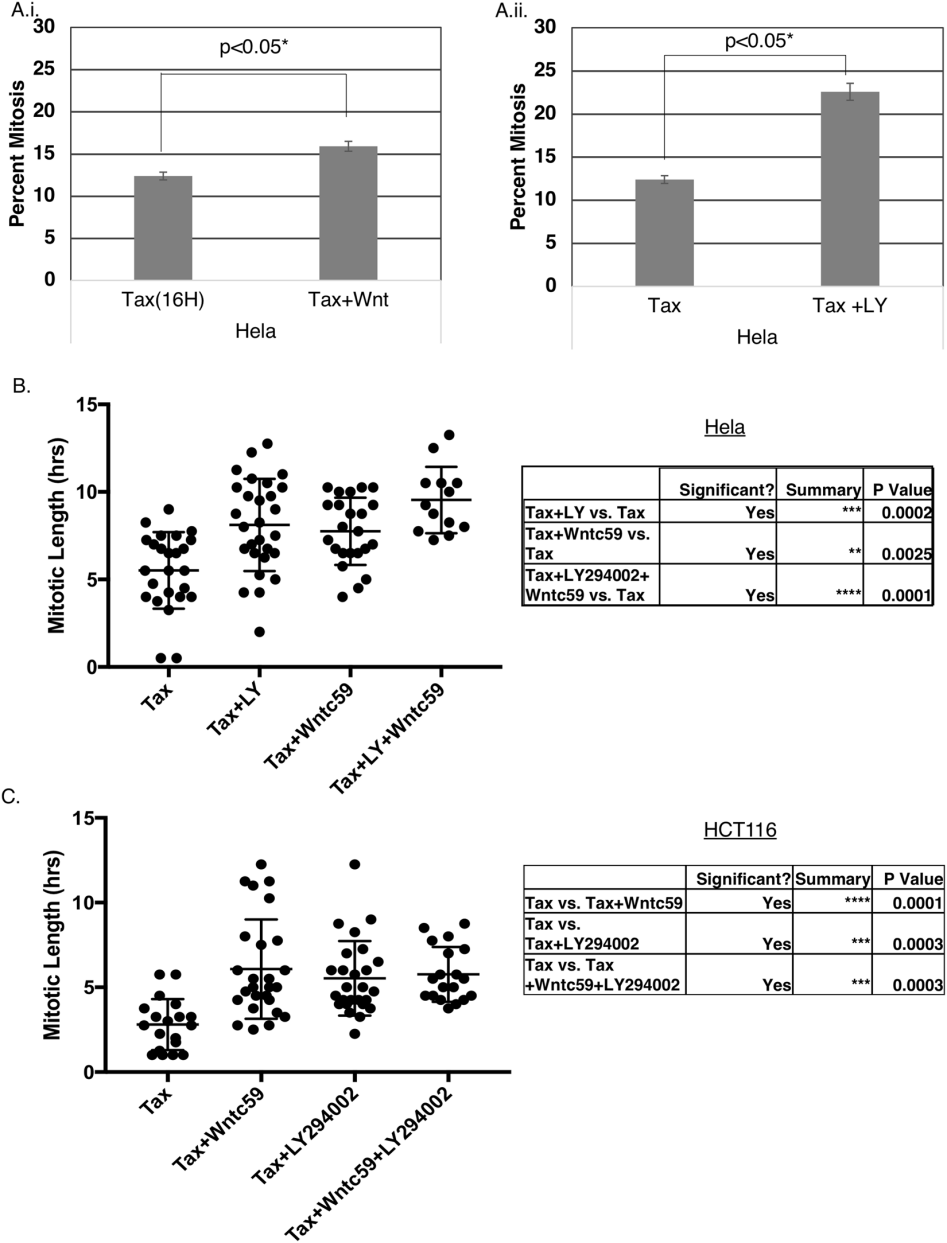
PI3K and WNT inhibitors induce a mitotic delay. (**A**,**B**) HeLa M cells were synchronized by single thymidine block and then exposed to 100 nM Taxol and either 2.8 μM LY294002 (LY) or 400 nM WNTc59. Cells were harvested after 13 hours in HCT116 or 16 hours in HeLa cells, and chromosome spreads used to analyze mitotic index. ~300 cells per condition was counted in a blinded manner, and the experiments performed in triplicate and repeated three times. (**C**) HeLa M cells or HCT116 cells (**D**) were synchronized by single thymidine block and then exposed to 100 nM Taxol and either 2.8 μM LY294002 (LY) or 400 nM WNTc59, or both and traced using time lapse analysis. Mitotic length was measured using mitotic entry and exit times, with 30 cells being counted for each treatment.

GSK3 is a multifunctional kinase pivotal in multiple pathways that regulate a variety of biological processes. Importantly, a novel WNT/STOP pathway has been implicated in regulating G2/M phase, where activation of the WNT pathway inhibits GSK3-dependent proteasomal degradation of multiple mitotic proteins^53^. There is also mounting evidence of PI3K/PTEN/Akt/mTOR influencing mitosis^39,40^. We thus investigated the influence of these upstream regulatory pathways on GSK3 regulation of the mitotic checkpoint. HeLa M cells were synchronized by single thymidine block and then exposed to 100 nM Taxol plus the PI3K inhibitor LY294002 (LY), or the WNT-signaling inhibitor WNTc59. The lower concentration of Taxol 100 nM allows cells to undergo a higher level of mitotic slippage, compared to Taxol 1 μM, providing an opportunity to test whether inhibition of upstream pathways may strengthen the arrest (our unpublished data). Mitotic index was modestly increased when HeLa M cells were exposed to the Taxol in combination with WNTc59 (Fig. 8A). Inhibiting PI3K increased mitotic arrest in low Taxol concentration more effectively than the WNT inhibitors (Fig. 8B). Next, synchronized HeLa (Fig. 8C) or HCT116 (Fig. 8D) were exposed to 100 nM Taxol together with LY and WNTc59 separately or together, and then followed by time lapse imaging. Addition of the upstream regulator inhibitors, LY or WNTc59, resulted in an increase in mitotic length compared to control cells. The increase in mitotic length was more pronounced when both compounds were added together. To analyze if the WNT- and PI3K- induced mitotic arrest was occurring via GSK3β, we measured mitotic length in cells treated with Taxol, WNTc59 and LY, with and without SB. Addition of SB to WNTc59 and LY treated cells resulted in shorter mitosis. These cells are treated with Taxol at 100 nM which causes the cells to undergo mitotic slippage, and thus the mitotic length is shorter than 1 μM Taxol (Supplemental Fig. 8). This indicates that the WNT- and PI3K- signaling pathways may be acting via GSK3 to regulate mitotic checkpoint.

## Discussion

We investigated the effect of small molecule kinase inhibitors on mitosis and found a novel role for GSK3 in mitotic regulation. Previous studies showed that treatment with GSK3 inhibitors alone induces chromosome misalignment, a condition that should trigger the mitotic checkpoint and cause a mitotic block. However, in these studies, despite causing a mitotic delay, GSK3 inhibitor-treated cells underwent anaphase with mis-aligned chromosomes^38^. This observation suggests that GSK3 plays a role in mitotic checkpoint strength. Consistent with this hypothesis, our data show that inhibiting GSK3 induces multiple cancer cell lines to exit mitosis in the presence of spindle toxins. We observed this effect with multiple, structurally distinct GSK3 inhibitors. Kinase inhibitors are most useful in cell cycle studies since they allow rapid effects to be achieved. However, off-target effects are a potential weakness that can limit the interpretations drawn from these compounds. Where possible we have followed up using RNAi or knock-out systems to strengthen the conclusion that GSK3 regulates the strength of the mitotic checkpoint. The GSK3β knockout MEFS showed a decreased mitotic index compared to wild type cells, confirming the effect was due to GSK3β. GSK3 has two isoforms β and α, which have redundancy in structure but very distinct biological functions. In general, GSK3 inhibitors inhibit both isoforms similarly.

In prolonged mitotic arrest induced by spindle toxins, the mitotic checkpoint gradually weakens, and the cells exit mitosis. When cells exit mitosis without aligning their chromosomes, this potentiates aneuploidy and may lead to chromosome instability. We show that GSK3 inhibition reduces recruitment of key checkpoint proteins to kinetochores and reduces MCC assembly, indicative of a weakened checkpoint^13,32,42^. GSK3 inhibition decreases levels of Mad2, BubR1 and Bub1 at the kinetochore in spindle-toxin –induced mitotic arrested cells. Additionally, GSK3 inhibition decreases MCC assembly, the core complex required for APC/C inhibition. GSK3β knockout MEFs also showed decreased levels of Mad2 and BubR1 at kinetochores. Similar results were obtained when GSK3β was reduced by RNAi in HeLa cells. Overall, these observations indicate that GSK3 enhances kinetochore recruitment of checkpoint proteins to ensure maximal mitotic arrest in cells exposed to spindle toxins. A number of critical checkpoint proteins recruited to unattached kinetochores trigger the assembly of MCC which inhibits APC/C activity to arrest cells in mitosis^16,54,55^. An active MCC is composed of two subcomplexes of Cdc20-Mad2 and BubR1-Bub3, giving rise to the core tetrameric MCC complex, which can inhibit APC/C activity^3^. BubR1-Bub3 and cMad2 can directly bind to Cdc20 and inhibit APC/C independently. However, BubR1 and Mad2 synergistically inhibit APC/C by enhancing each other’s binding to Cdc20^2^. GSK3 –dependent decrease in Mad2 regulation not only indicates a novel regulatory mechanism for APC/C activity and mitotic arrest, but also a novel regulatory mechanism of mitotic checkpoint strength. As an interplay between pro-apoptotic signals and Cyclin B levels decide cell fate in prolonged mitotic death in anti-mitotic drugs, GSK3 regulation of the mitotic checkpoint strength may introduce a new player in this relationship^56^.

We currently do not know how GSK3 regulates the mitotic checkpoint, but it is possible that direct phosphorylation of kinetochore-proximal proteins enhances recruitment of Mad2, BubR1 and Bub1. Related to this idea, GSK3 regulates spindle morphology and microtubule dynamics. It modulates microtubules in part by regulating the activity of microtubule associated proteins (MAPs) such as CLASP2^57,58^. In this case, phosphorylation of CLASP2 reduces its binding to microtubule ends near kinetochores. Further, expressing a phoshphomimic form of CLASP2 decreased interkinetochore distance ~10% compared to controls suggesting that phosphorylation may decrease kinetochore-microtubule interactions. Since, microtubule attachment triggers recruitment of checkpoint proteins to kinetochores, CLASP2 is a GSK3 target that warrants further investigation with respect to the mitotic checkpoint.

Inhibiting GSK3 overcomes the mitotic arrest induced by compounds that stabilize microtubules. The fact that GSK3 is a critical signal transduction protein suggests that under some circumstances, extracellular signals may modulate mitotic checkpoint. Indeed, other components in the WNT pathway have roles in mitosis regulation^34,35,59,60^. One important implication of this idea is that cancer cells in which growth factor pathways are activated may show altered responses to chemotherapeutic agents that target the spindle. Our experiments with inhibitors of WNT and PI3K signaling indicate that these pathways can regulate the strength of the mitotic checkpoint possibly by regulating GSK3.

## Methods

### Cell culture, synchronization and drug treatment

Cells were grown in Dulbecco’s minimal essential medium (Gibco) with penicillin/streptomycin and 10% fetal bovine serum in a humidified atmosphere of 10% CO_2_ at 37 °C. Experiments were performed with HeLa M cells, a sub-line of HeLa. HCT116 cells were obtained from Dr. B. Vogelstein^61^, HT-1080 fibroblast cells were from Dr. G.R Stark and wild type and gsk3−/− mouse embryo fibroblast (MEF) were obtained from Dr. Jim Woodgett^62^. Cells were synchronized by treating with 2 mM thymidine (Sigma Aldrich) for 20–21 hours for HeLa cells and 24 hours for HCT116 cells, followed by release for 9–10 hours before adding respective drugs.

Taxol (100 nM or 1 μM) (Cayman Chemical), Epothilone (20 nM) (Sigma-Aldrich) or Nocodazole (2 μM) were added to arrest cells in mitosis, after thymidine release where mentioned. The proteasome inhibitor MG132 (Cayman chemical) was used at a concentration of 20 μM and kinase inhibitors were used at previously determined effective concentrations: 2.5 μM ZM447439 (AstraZeneca), 2 μM Reversine (Calbiochem), 2.8 μM LY204002 (Cayman Chemicals), 30 μM SB415286 (Cayman Chemicals), 400 nM WNTc59 (Cayman Chemicals), RO 318220 (Cayman Chemicals), 60 mM Lithium Chloride, 14 μM RO3306 (Cayman Chemicals) and 10 μM Chelerythrine Chloride (Cayman Chemicals). Finally, cell selection was accomplished using puromycin at 2 μg/μL (Invitrogen).

### Immunofluorescence microscopy

For immunofluorescence microscopy, cells were grown on coverslips. Following transfection and selection and/or drug treatments, cells were fixed with 2% formaldehyde in PBS for 10 minutes and then permeabilized with 150 mM NaCl, 10 mM Tris (pH 7.7), 0.1% Triton-X-100 (v/v) and 0.1% BSA (w/v) for 9 minutes^63^. Fixed cells were blocked with PBS containing 0.1% BSA and 0.02% sodium azide for 16 hours at 4 °C.

Detection with the following primary antibodies were done where indicated: rabbit anti-Mad2 (A300-301A, Bethyl), rabbit anti-BubR1 (A300-386A-M; Bethyl), rabbit anti-Aurora B (H75; Santa Cruz), mouse anti-INCENP (58-217; Millipore), human anti-ACA (CREST, Antibodies Inc.) rabbit FITC conjugated anti-V5 (C29F4; Cell signaling), rabbit anti-GSK3β (D5C5Z; Cell Signaling) mouse anti-GFP (3E6; Invitrogen) or rabbit anti-Bub1 (Gift from Dr. Song-Tao Liu). Conjugated secondary antibodies included goat-anti-rabbit Alexafluor-488, goat-anti-rabbit Alexafluor-568, goat-anti mouse Alexaflour-568 and goat-anti-human Alexafluor- 633.

Antibodies were generally diluted at either 1:100, 1:200, 1:500 or 1:2000 in PBS containing 0.1% BSA and 0.02% sodium azide. Coverslips were mounted using 1% p-Phenylenediamine dihydrochloride, 10 mM Tris pH 9.0, 90% glycerol and analyzed using a Leica SP8 confocal microscope. Individual kinetochores were imaged by successive acquisition of 2.5 μm volumes at 1.0 μm per step along the Z axis at a zoom factor of 4–5. Images are generally projections of multiple planes.

For quantification of immunofluorescence measurements, while taking images the intensity and laser power were all kept constant. During analysis and quantification, first the control, for example ACA, was used as a control to delineate the KT, and then the protein of interest, example Mad2, BubR1,Bub1, Knl1 and Aurora B levels in that marked KT area was measured. When quantifying, intensities of a specific protein were compared between treated and untreated for that specific experiment.

### Chromosome drops

Cells were transfected with GSK3-V5 tagged plasmid where stated and then blocked in mitosis with Taxol. Cells were trypsinized and harvested in DMEM without FBS and P/S. Cell pellets were exposed to 0.075 M KCl to swell the cells, and fixed with methanol:acetic acid (3:1 v/v)^64^. The cells were dropped onto slides, and stained with Giemsa (264983, Protocol) diluted 1:1 with dH_2_O, for 5 five minutes and then rinsed three times in dH_2_O. Following this, the slides were air-dried and a drop of Vectamount (SO509, Vector) was placed on the cells and covered with coverslip. Chromosome morphology and mitotic index was determined for each sample in a blinded manner and included data from at least three independent experiments performed in triplicate. Representative images are shown in Supplemental Fig. 10B.

### Live cell imaging

Cells growing in 24-well plates were synchronized by a single thymidine block. Cells were released 20–21 hours later into Hams F12 media and allowed to grow for an additional 9 hours. HEPES-KOH (pH 7.6) was added at 0.3 units/ml (20 mM) and then the medium was carefully covered with a layer of mineral oil. Live cell imaging of HeLa M cells was performed similarly as before^65,66^ on an automated Olympus IX-81 microscope to collect phase contrast and GFP (using Alexa Fluor 488 channels) images at 15 minute intervals using a 20×, NA 0.50 UPlanFLN objective while cells were maintained at 37 °C in a heated chamber.

Drugs were added to the medium immediately before imaging, unless where stated that Taxol was added prior. Phase-contrast and GFP images were collected on an automated Olympus IX-81 microscope using a 20×, NA 0.50 UPlanFLN objective.

### Cell lysates, immunoblotting and co-immunoprecipitation

Cells were scraped into phosphate buffered saline (PBS) followed by centrifugation (16000 *g*, 4 °C) for 5 min and stored at −80 °C. Pellets were lysed with RIPA Buffer (10 mM Tris [pH 7.4], 150 mM NaCl, 1% NP-40, 1% DOC, 0.1% SDS) supplemented with 1 μg/ml aprotinin, 2 μg/ml leupeptin, 1 μg/ml pepstatin A, 1 mM dithiothreitol, 0.1 M phenylmethyl sulfonyl fluoride, 1 mM sodium fluoride, and 1 mM sodium vanadate for 30 minutes on ice and centrifuged at maximum speed for 35 minutes at 4 °C. Protein concentration of each lysate was determined by using the BCA Protein Assay Kit (Pierce).

Immunoblotting was used to probe specific proteins in the cell lysates and immunoprecipitated. In some experiments the blots were scanned, and the intensities of bands of interest were quantified using ImageJ software.

For immunoprecipitation, 200–300 μg of lysates were incubated with appropriate antibodies (0.5–1 μg) at 4 °C for 4 hours and then mixed with protein A-agarose beads (RepliGen) for 1 hour. Immune complexes were washed four times with cell lysis buffer containing 250 mM NaCl and then subjected to western blotting.

Equal protein amounts were separated by sodium dodecyl sulfate polyacrylamide gel electrophoresis (SDS-PAGE) and transferred to polyvinylidene difluoride membranes (Millipore) in Transfer Buffer (25 mM Tris, 192 mM glycine, 10% methanol). The membranes were blocked for 1 hour with PBST [1× PBS plus 0.05% Tween 20] containing 5% non-fat dry milk. Membranes were probed with Ark2 (1:400 dilution), Borealin (elution), GSK3β (1:5000) β-catenin (1: 1000, D10A8, Cell Signaling), Mad2 (1:1000), BubR1 (1;1000), Cdc20 (sc-13162, Santa Cruz Biotechnology, 1:200), Cdc27 (1:1000) and actin (1:1000) antibodies from Invitrogen (diluted 1:2500) followed by secondary antibodies from Biorad diluted 1:10000. Antibody binding was detected using the Clarity chemiluminescence substrate (Biorad)^67^.

### siRNA

To deplete GSK3β, SMARTpool siRNA against GSK3B (2932) was used in all experiments at a final concentration of 80 nM (Dharmacon ON-TARGETplus Human GSK3β (L-003010-00-0005). Dharmacon scrambled siRNA was used as controls at 80 nM. siRNA transfection was performed using Lipofectamine 3000 (Invitrogen) per manufacturer’s instructions.

### DNA transfection and plasmids

DNA transfection was carried out using polyethylenimine (PEI) following a modified protocol^68,69^. Briefly, linear PEI (MW 25,000, from Polysciences) was dissolved in 0.2 N HCl at 1 mg/ml (final pH around 1.0) for long term storage at −80 °C. For transfection, the thawed PEI was neutralized to pH 7.0 with NaOH and used at a DNA:PEI mass ratio of 1:3.

### CRISPR gene knockout

CRISPR technology to create GSK3β knockout human cell lines were carried out using a modified protocol^70^. Briefly, 2 sets of guideRNAs were designed against GSK3β at exon5, gRNA top… 5′CACCGATCCATTCCTTTGGAATCTGCCATCGGGAT′3 and gRNA bottom…5′AAACATCCCGATGGCAGATTCCAAAGGAATGGATC′3, and exon 6, gRNA top… 5′CACC-GATGTTTCGTATATCTGTTCT′3 and gRNA bottom… 5′AAAC-AGAACAGATATACGAAACATC′3. The first gRNA set was cloned into a plasmid containing the Cas9 and the sgRNA scaffold pSpCas9BB-2A-puro (Addgene) and the second gRNA set was cloned into a plasmid containing Cas9 and the sgRNA scaffold pSpCas9BB-2A-GFP. The two plasmids were transfected into HeLa cells using PEI as previously described, and cells were subjected to 2 μg/μl puromycin selection for three days, as well as looking for GFP positive cells prior to selection.

gRNAs against IL-17A were used to construct a CRISPR plasmid and used as control. The gRNAs used were gRNA top… 5′CACCG-GGTTGACCATCACAGTCCGG′3 and gRNA bottom 5′AAAC-CCGGACTGTGATGGTCAACC-C′3. (or gRNA top… 5′CACCG-GAGAAGATACTGGTGTCCGT′3 and gRNA bottom… 5′AAAC-ACGGACACCAGTATCTTCTC-C′3).

## Electronic supplementary material

Fig 1-11
