# Supplementary material for: Analysis of the role of GSK3 in the mitotic checkpoint
Source: Sci Rep. 2018 Sep 24;8:14259. doi: 10.1038/s41598-018-32435-w (PMC6155330; doi:10.1038/s41598-018-32435-w)
Supplement: Supplementary file 1 — Fig 1-11 [file 41598_2018_32435_MOESM1_ESM.pdf]

Analysis of the role of GSK3 in the mitotic checkpoint.

Rashid, MS, Mazur, T, Ji, W, Liu, ST, and *Taylor, WR.

Department of Biological Sciences, University of Toledo, 2801 W. Bancroft Street, MS601, Toledo, OH 43606
Address correspondence to WRT: Phone (419) 530-1966, FAX (419) 530-7737, Email: william.taylor3@utoledo.edu

Supplemental Figure 1

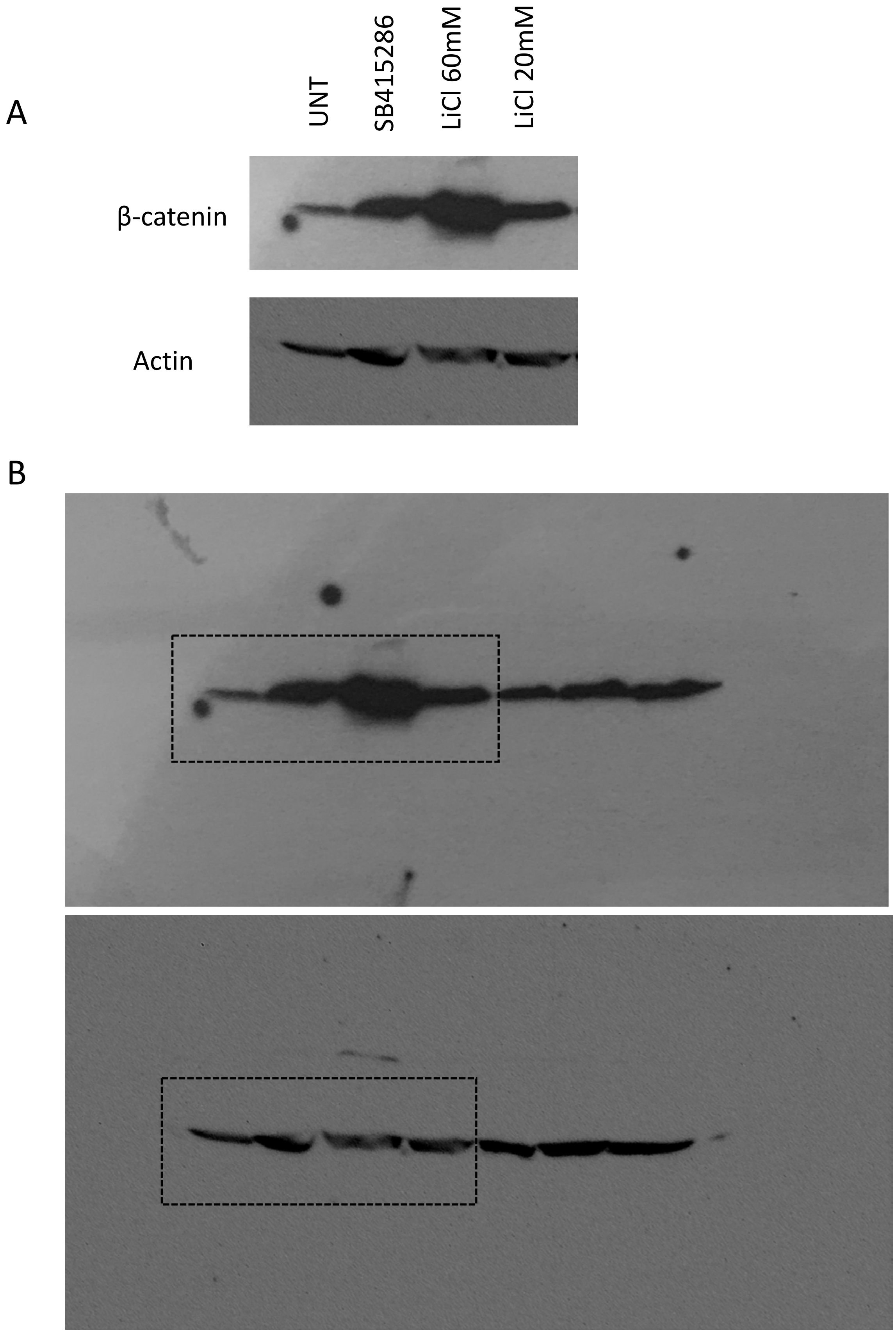

Supplemental Figure 1. GSK3 inhibitors show increase in β-catenin in Hela cells. (A) GSK3 inhibitors, SB or RO and were added for 2.5h and then cells were analyzed by western blot for b-catenin. Actin was used as loading control. (B) Full blots of experiment

Supplemental Figure 2

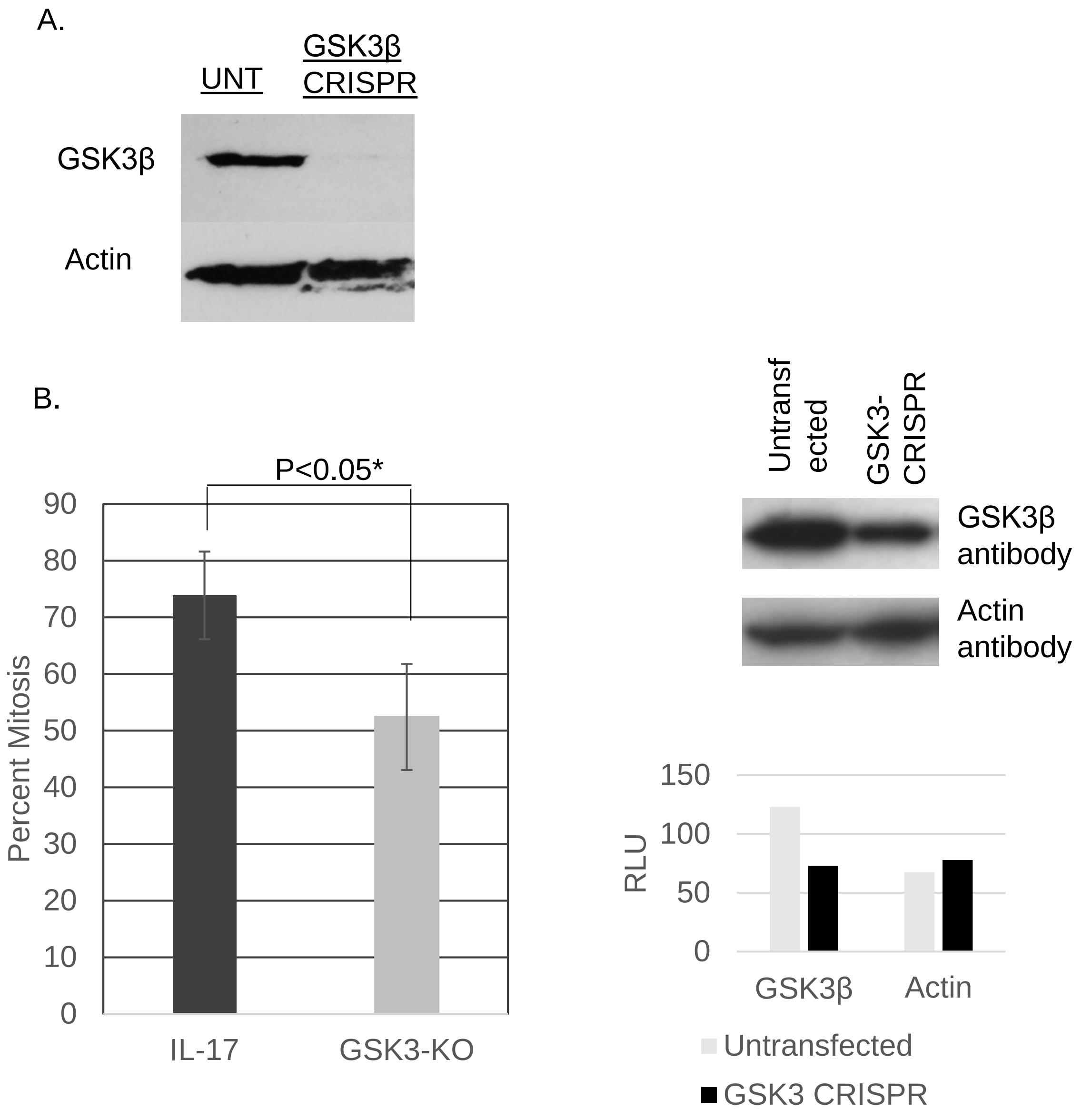

Supplemental Figure 2. GSK3 CRISPR expressed transiently showed a decrease in mitotic index. **(**A) The GSK3 CRISPRs were transfected into cells, subjected to puromycin, and then expanded to obtain stable colonies. Colonies were picked and cultured and then cells harvested for western blot analysis. (B) Two sets of gRNAs were designed against GSK3b exon3 and exon5 and co-transfected into cells. One set was cloned into a CRISPR/Cas-9 plasmid containing puromycin resistance and the other containing a GFP plasmid. Following a 24-hour transfection cells were subjected to a two day of higher puromycin and then washed for a day and then another round of lower puromycin. Cells were also mock transfected with CRISPR plasmid containing IL-17 gRNAs. Cells were then synchronized with single thymidine block and exposed to Taxol and chromosome spreads done to obtain mitotic index.

Supplemental Figure 3

C

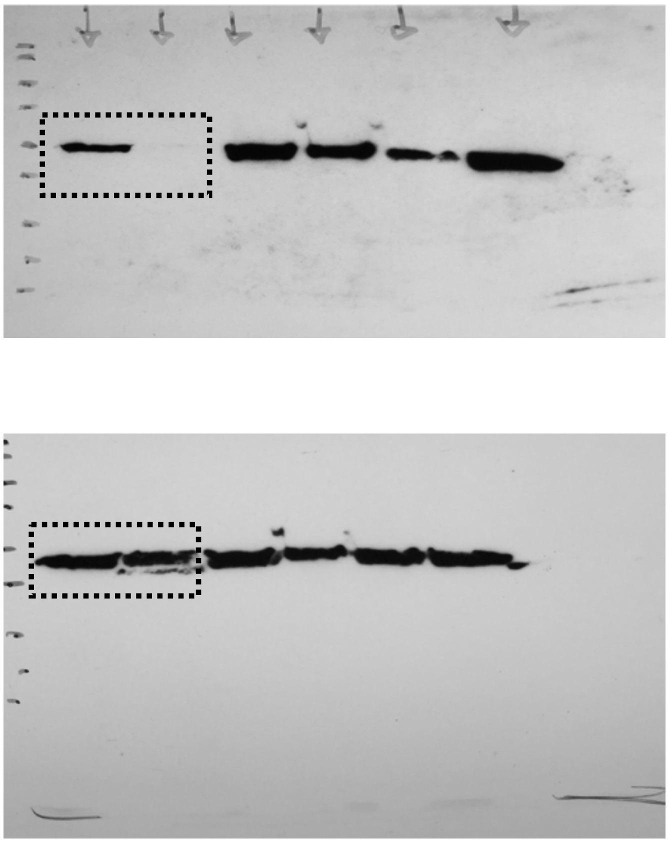

D

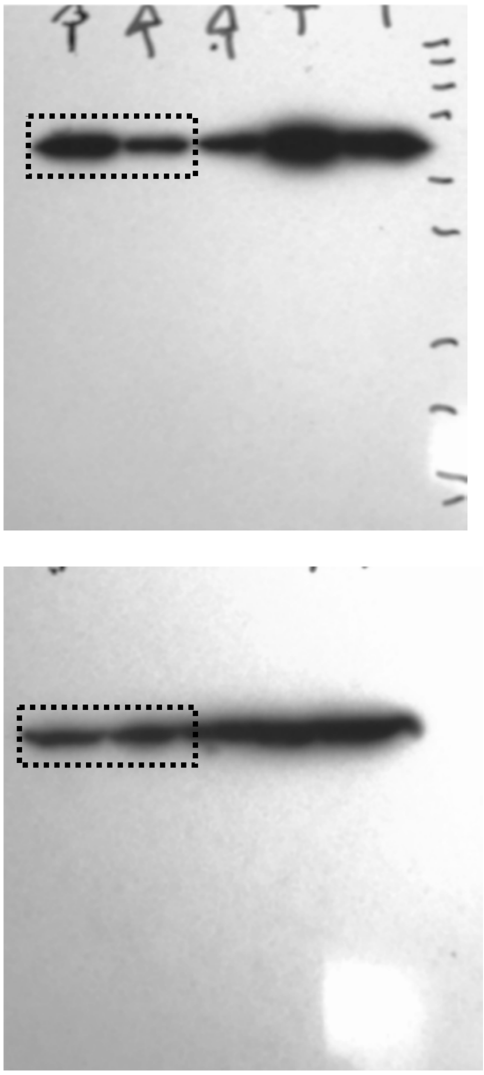

Supplemental Figure 3. GSK3 CRISPR expressed transiently showed a decrease in mitotic index. **(**C) Full blot of supplementary figure 2A. (D) Full blot of supplementary figure 2B.

Supplemental Figure 4

A

GSK3β

Actin

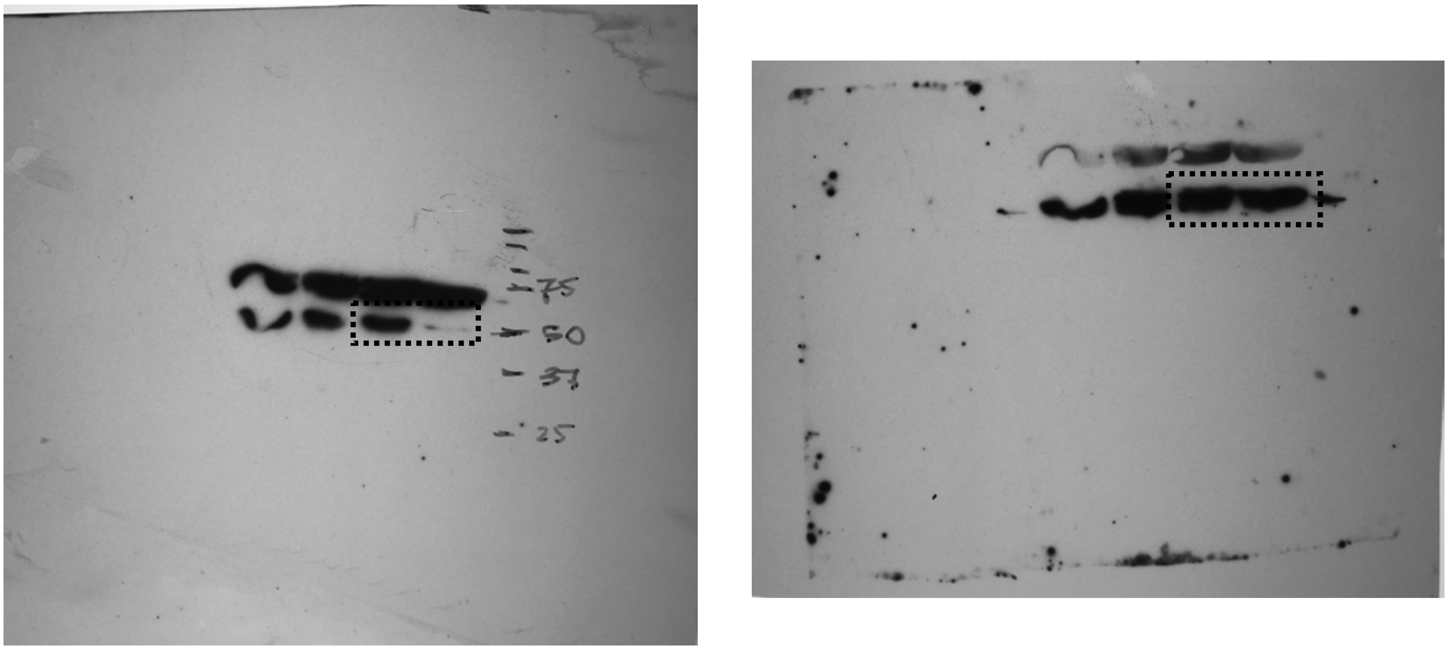

B

gsk3-/- MEFs

WT MEFs

GSK3β

Actin

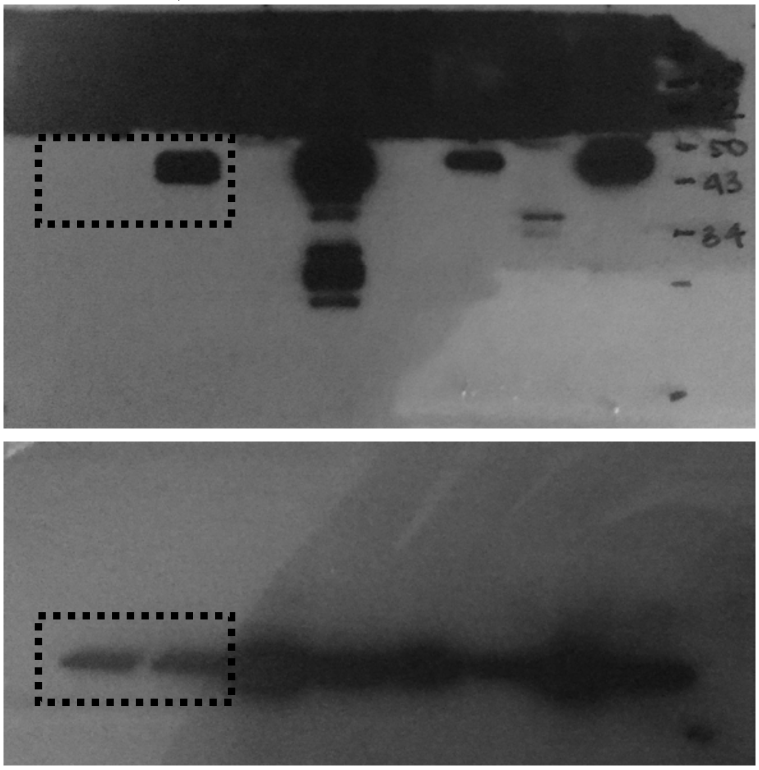

Supplemental Figure 4. (A)Full blots of Figure 3B. (B) WB showing GSK3β, and actin control, in WT and gsk3β-/- MEFs.

Supplemental Figure 5

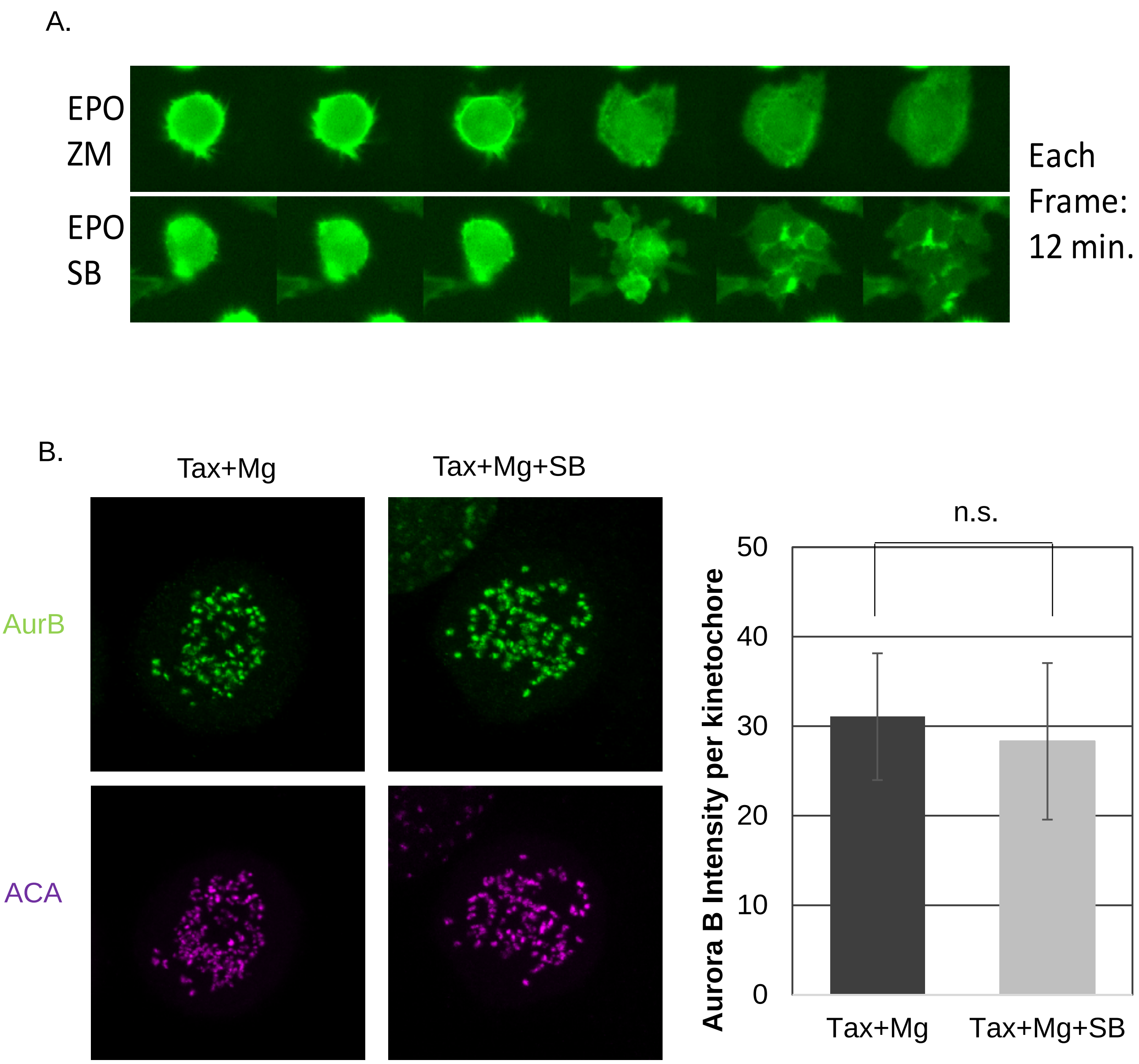

Supplemental Figure 5. Formation of cleavage furrows in cells exiting mitosis after Gsk3 inhibition. (A) HeLa cells were transfected with Lifeact to mark actin in live cells, exposed to epothilone B for 20 hours and then treated with either ZM to inhibit Aurora B or SB to inhibit Gsk3. Time-lapse microscopic frames are shown. By morphology, both cells appear to be in mitosis in the first frame shown. (B) HeLa cells were exposed to 1mM Taxol with or without SB. Mg132 was added to inhibit the proteasome. Cells were subjected immunofluorescence analysis after 2.5H and analyzed for Aurora B levels, ACA, a kinetochore marker, and DAPI to stain chromosomes. ~10 cells per condition were imaged using confocal, using maximum projection images of z-stacks to analyze antigen levels at kinetochores. ~300 kinetochores were analyzed for each condition, using image J software to obtain intensity of the indicated antigens at kinetochores.

Supplemental Figure 6

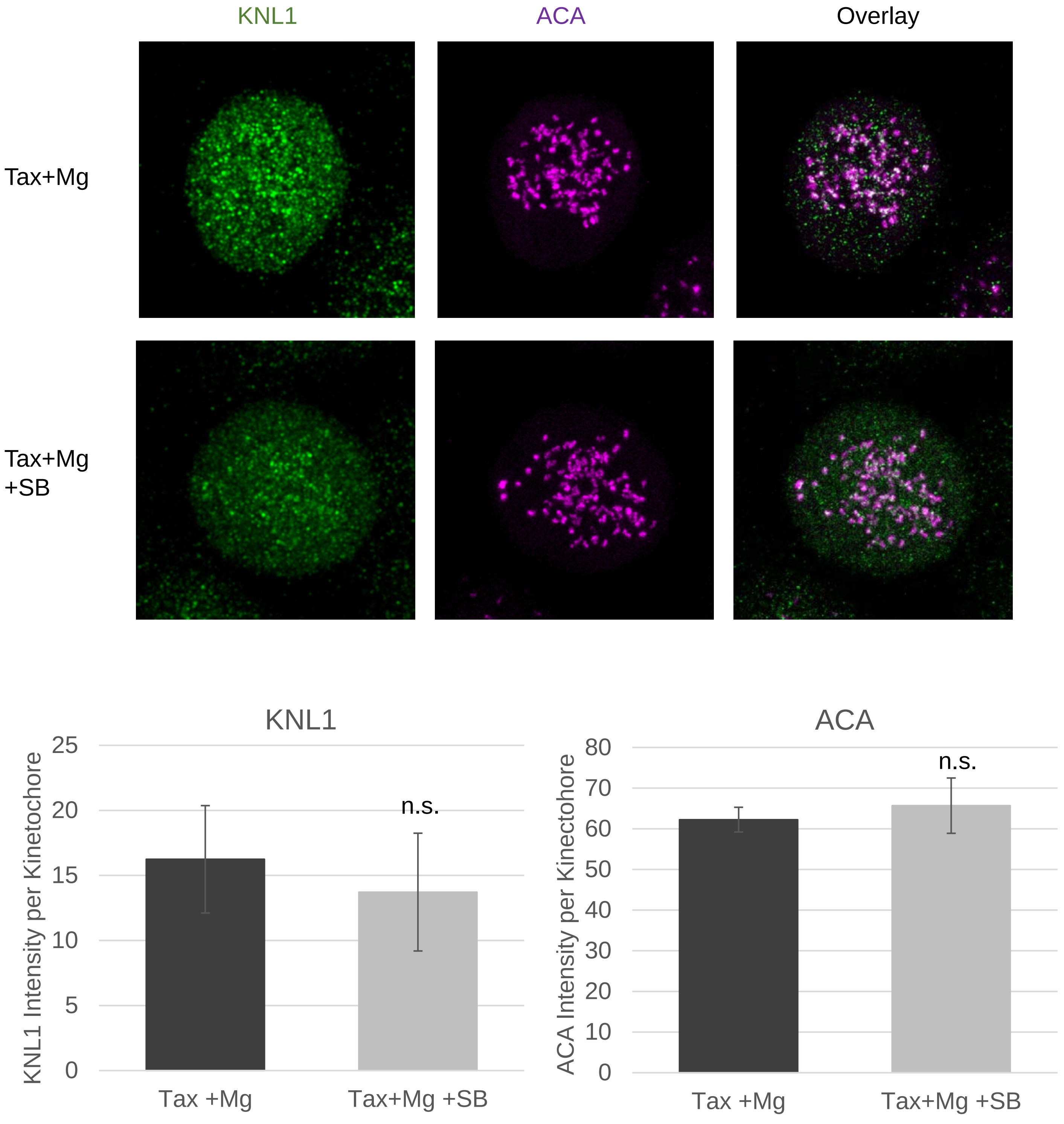

Supplemental Figure 6. Knl1 levels remain unchanged at kinetochores when exposed to GSK3 inhibitor in the presence of spindle toxins. HeLa cells were exposed to 1mM Taxol with or without Sb (30mM) or DMSO control. The cells were then subjected to immunofluorescence analysis for Knl1, ACA, and DAPI to stain chromosomes. Mg132 was added to inhibit the proteasome. ~10 cells per condition were imaged using confocal, using maximum projection images of z-stacks to analyze antigen levels at kinetochores. ~300 kinetochores were analyzed for each condition, using image J software to obtain intensity of the indicated antigens at kinetochores.

Supplemental Figure 7

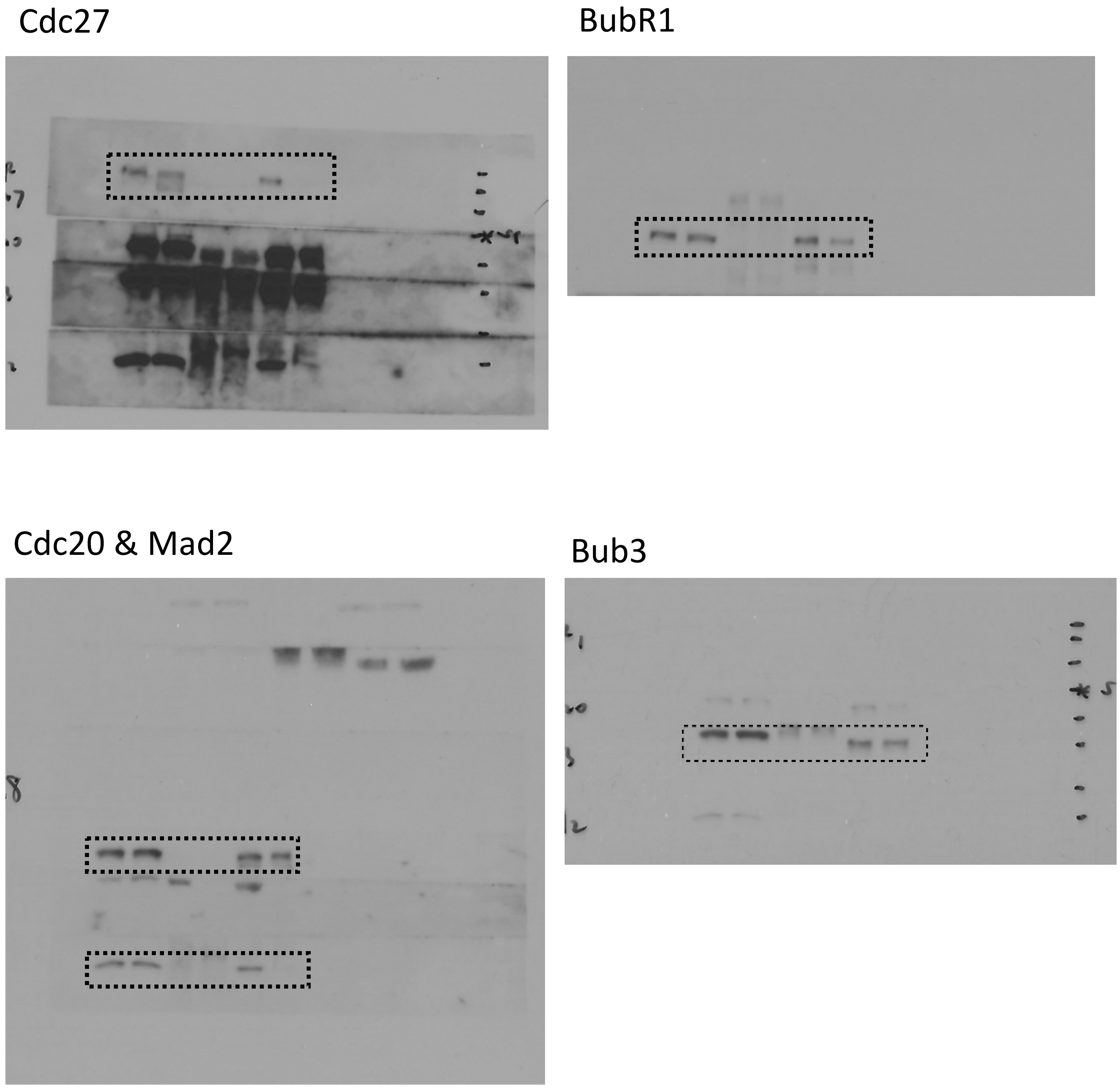

Supplemental Figure 7. Full blots of Figure 6. GSK3 inhibition decreases MCC assembly in the presence of spindle toxins. HeLa cells were exposed to 1μM Taxol for 12h, following which DMSO control or SB was added. Mg132 was added simultaneously. Cells were harvested after 2.5 hours and lysed. Co-immunoprecipitation was performed with extracted proteins pulled down using BubR1 antibody and IgG, as control, and 10% WCE was as used as input. Blots were striped and re-probed for different primary antibodies.

Supplemental Figure 8

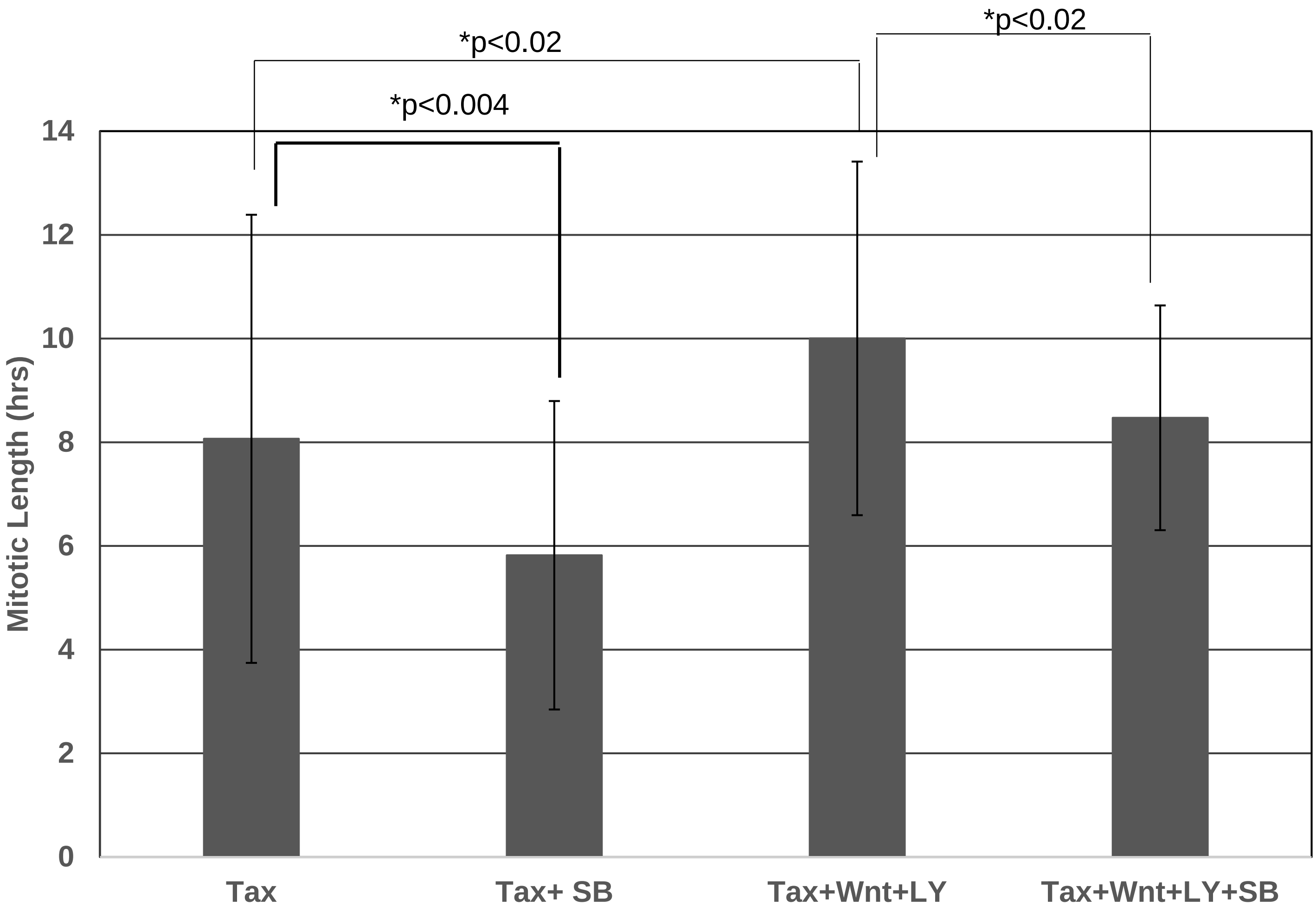

Supplemental Figure 8. GSK3 inhibitor shortens PI3K- and WNT- inhibitors induced mitotic delay. HeLa M cells were synchronized by single thymidine block and then exposed to 100nM Taxol alone or with both 2.8mM LY294002 (LY) or 400nM WNTc59. The cells were followed by phase-contrast time-lapse imaging. 13H later SB was added and the same cells were followed. ~40 cells were analysed per experiment. Mitotic entry and mitotic exit times were used to measure mitotic length.

Supplemental Figure 9

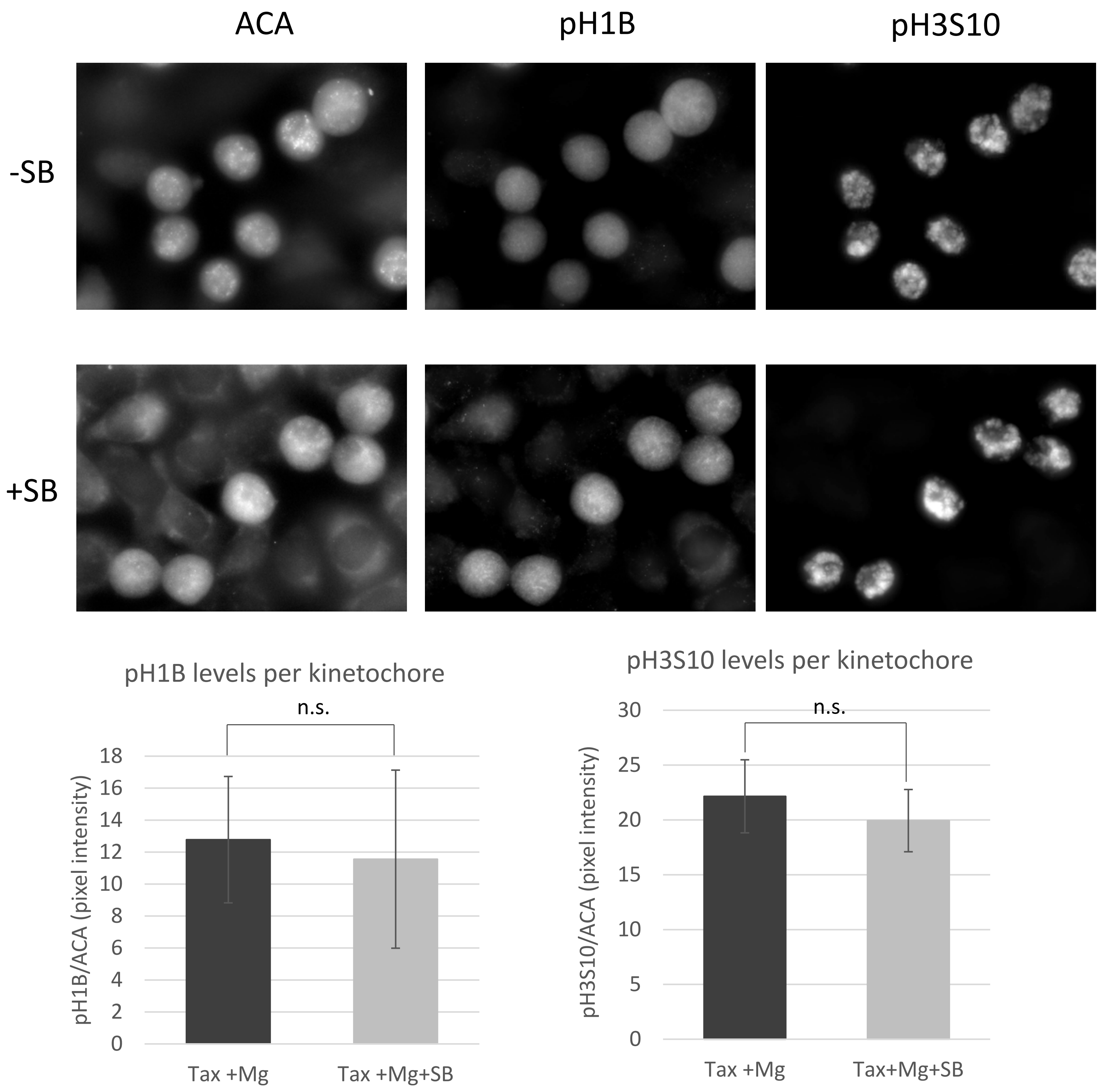

Supplemental Figure 9. GSK3 inhibitor does not affect CDK1 and Aurora B kinase activity. HeLa M cells were treated with Taxol 1µM and then 12 hours later followed by SB415286 30µM or DMSO control. Mg132 was added to inhibit the proteasome. ~2.5 hours later immunofluorescence analysis was done using antibody against phosphorylated histone H3, phosphorylated histone 3 serine 10 or ACA. ~10 cells per condition were imaged using confocal, using maximum projection images of z-stacks to analyze antigen levels at kinetochores. ~300 kinetochores were analyzed for each condition, using image J to obtain intensity of the indicated antigens at kinetochores

Supplemental Figure 10

A.

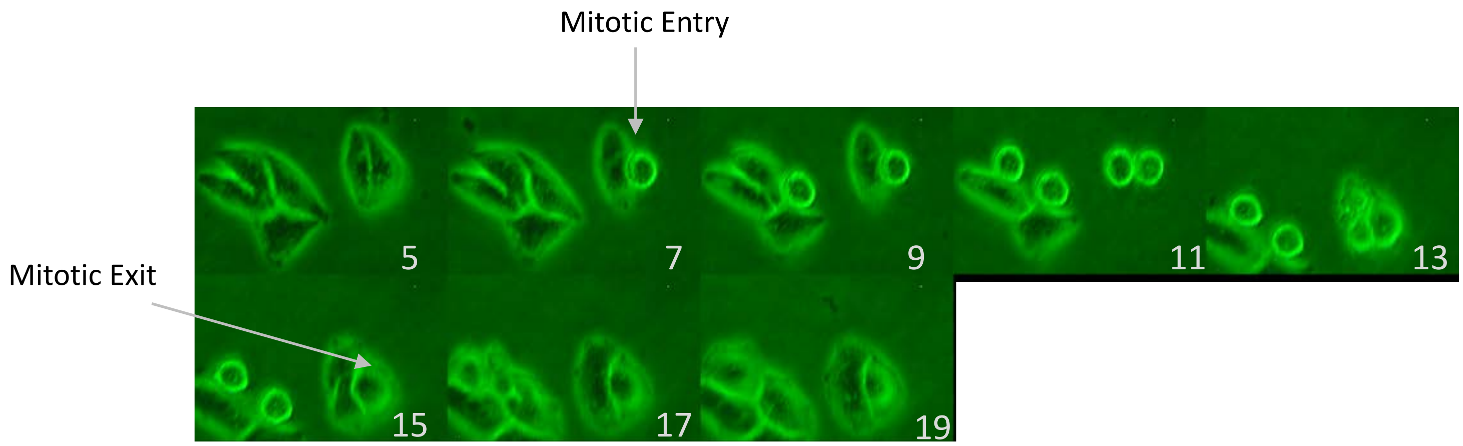

Inlet: Time (H) post-addition of Taxol. LiCl was added 11 hours post addition of Taxol.

B.

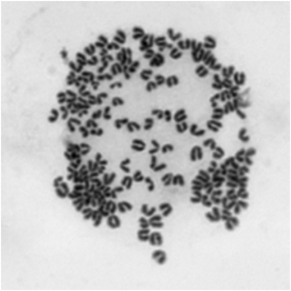

Mitotic cell

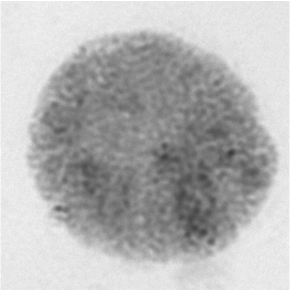

Interphase cell

Supplemental Figure 10. Representative mitotic entry and exit cells used for Time-lapse imaging and Chromosome drop experiments to measure mitotic index and mitotic length.

Supplemental Figure 11

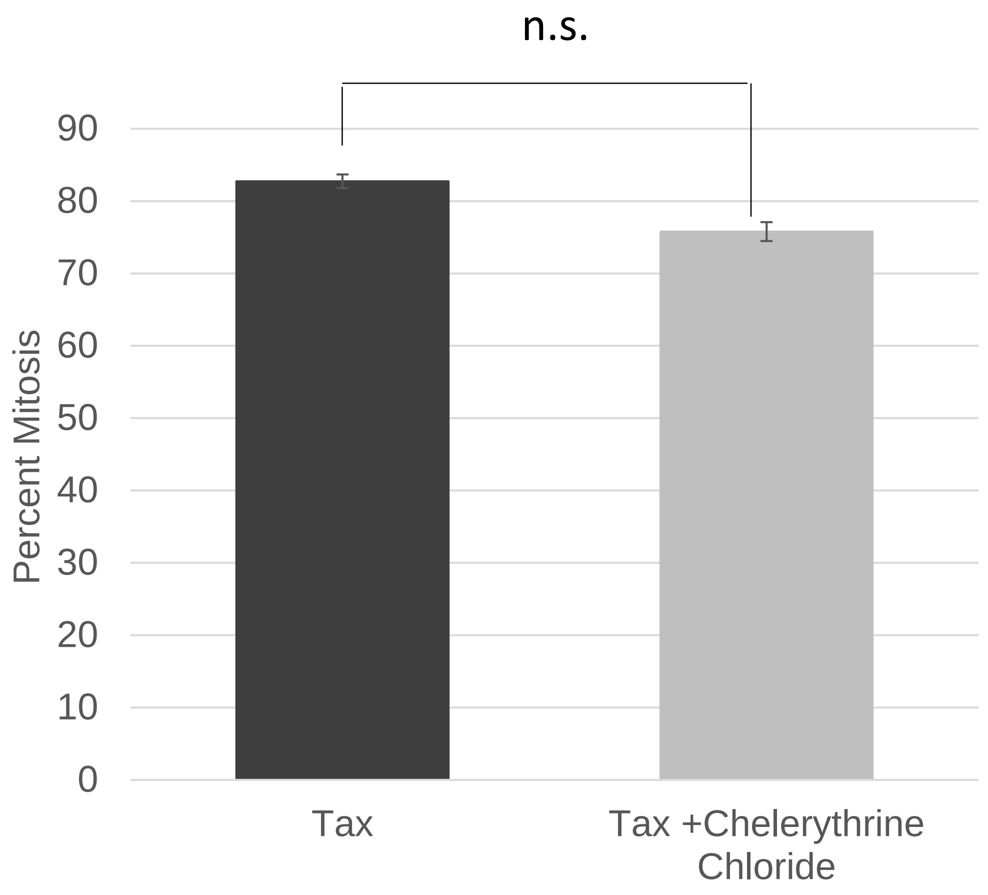

Supplemental Figure 11. PKC inhibition does not induce mitotic exit in Taxol arrested mitotic cells. Hela cells were exposed to 1µM Taxol for 12hrs. Following this 10µM Chelerythrine Chloride, PKC inhibitor, was added to the cells. 3 hrs later mitotic index in cells exposed to Taxol-only and Taxol+Chelerythrine Chloride was analyzed by chromosome spreads. 100 cells were counted in each experiment, and each treatment was done in triplicate.
